# Elucidating the Structure and Composition of Individual Bimetallic Nanoparticles in Supported Catalysts by Atom Probe Tomography

**DOI:** 10.1021/jacs.3c04474

**Published:** 2023-07-25

**Authors:** Florian Zand, Suzanne J. T. Hangx, Christopher J. Spiers, Peter J. van den Brink, James Burns, Matthew G. Boebinger, Jonathan D. Poplawsky, Matteo Monai, Bert M. Weckhuysen

**Affiliations:** † Inorganic Chemistry and Catalysis Group, Institute for Sustainable and Circular Chemistry and Debye Institute for Nanomaterials Science, Utrecht University, 3584 CG Utrecht, The Netherlands; ‡ High Pressure and Temperature Laboratory, Utrecht University, 3584 CB Utrecht, The Netherlands; § Shell Global Solutions, 1031 HW Amsterdam, The Netherlands; ∥ Center for Nanophase Materials Sciences, Oak Ridge National Laboratory, Oak Ridge, Tennessee 37831, United States

## Abstract

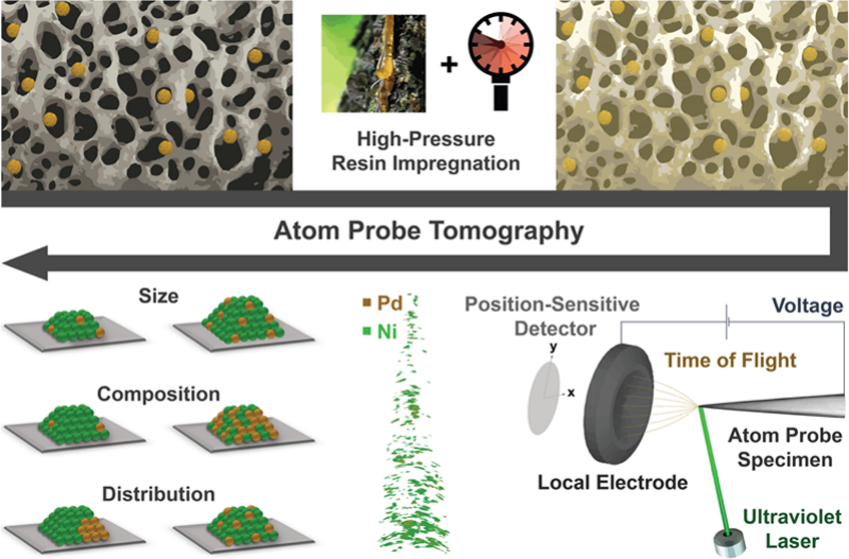

Understanding and controlling the structure and composition of nanoparticles in supported metal catalysts are crucial to improve chemical processes. For this, atom probe tomography (APT) is a unique tool, as it allows for spatially resolved three-dimensional chemical imaging of materials with sub-nanometer resolution. However, thus far APT has not been applied for mesoporous oxide-supported metal catalyst materials, due to the size and number of pores resulting in sample fracture during experiments. To overcome these issues, we developed a high-pressure resin impregnation strategy and showcased the applicability to high-porous supported Pd–Ni-based catalyst materials, which are active in CO_2_ hydrogenation. Within the reconstructed volume of 3 × 10^5^ nm^3^, we identified over 400 Pd–Ni clusters, with compositions ranging from 0 to 16 atom % Pd and a size distribution of 2.6 ± 1.6 nm. These results illustrate that APT is capable of quantitatively assessing the size, composition, and metal distribution for a large number of nanoparticles at the sub-nm scale in industrial catalysts. Furthermore, we showcase that metal segregation occurred predominately between nanoparticles, shedding light on the mechanism of metal segregation. We envision that the presented methodology expands the capabilities of APT to investigate porous functional nanomaterials, including but not limited to solid catalysts.

## Introduction

Large-scale industrial operations, such as pollution abatement technologies and base materials production, are reliant on effective catalyst materials. Within the family of materials, supported metal catalysts are a major group, as can be noted by the multitude of large-scale chemical processes for which they are applied.^1−5^ Optimizing supported metal catalysts has a significant impact on reducing energy demand, limiting waste formation, and maximizing material usage in such processes. Commonly this approach is guided by fundamental insights into structure–composition-performance relationships. In catalyst materials, size, shape, and composition of the metal nanoparticles are often strongly linked with their catalytic performance.^6−10^ This concept has been proven highly valuable to enhance catalytic properties beyond the capabilities of monometallic counterparts, as was shown for Pd–Ni-based bimetallics.^11−15^ However, achieving and maintaining a uniform optimal nanostructure is nontrivial, as metals are prone to maldistribution, interparticle migration, and Ostwald ripening on the medium scale (10–100 nm scale) and intraparticle segregation at the microscale (<10 nm) (e.g., core–shell vs homogeneous alloy). Such nonuniformity also is strongly related to the pore structure of the support, thus making the system very complicated.

While the size and shape of the individual metal nanoparticles can be successfully assessed by high-end electron microscopy techniques, high-resolution compositional information on a statistically relevant number of metal nanoparticles is still rather hard to obtain. Techniques, such as X-ray diffraction (XRD) and X-ray absorption spectroscopy (XAS), offer valuable insights into crystallographic phases, phase composition, oxidation states, and even coordination environments. Nevertheless, XAS and XRD results represent bulk averages and lack sensitivity, especially for nanosized alloys with very low concentrations. Furthermore, scanning transmission X-ray microscopy (STXM) enables spatially resolved compositional mapping even in combination with coordination environment probing, while resolutions are generally limited to around 10 nm.^16,17^ Recent advances in electron tomography (ET) combined with energy-dispersive X-ray spectroscopy (EDX) and electron energy loss spectroscopy (EELS) further expand our capabilities by gaining 3D compositional information even coupled with valence state mapping for EELS. Still, generally, only a small number of nanoparticles can be probed due to the time-consuming nature of ET, which complicates the assessment of compositional heterogeneities between metal nanoparticles.

**Figure 1:**
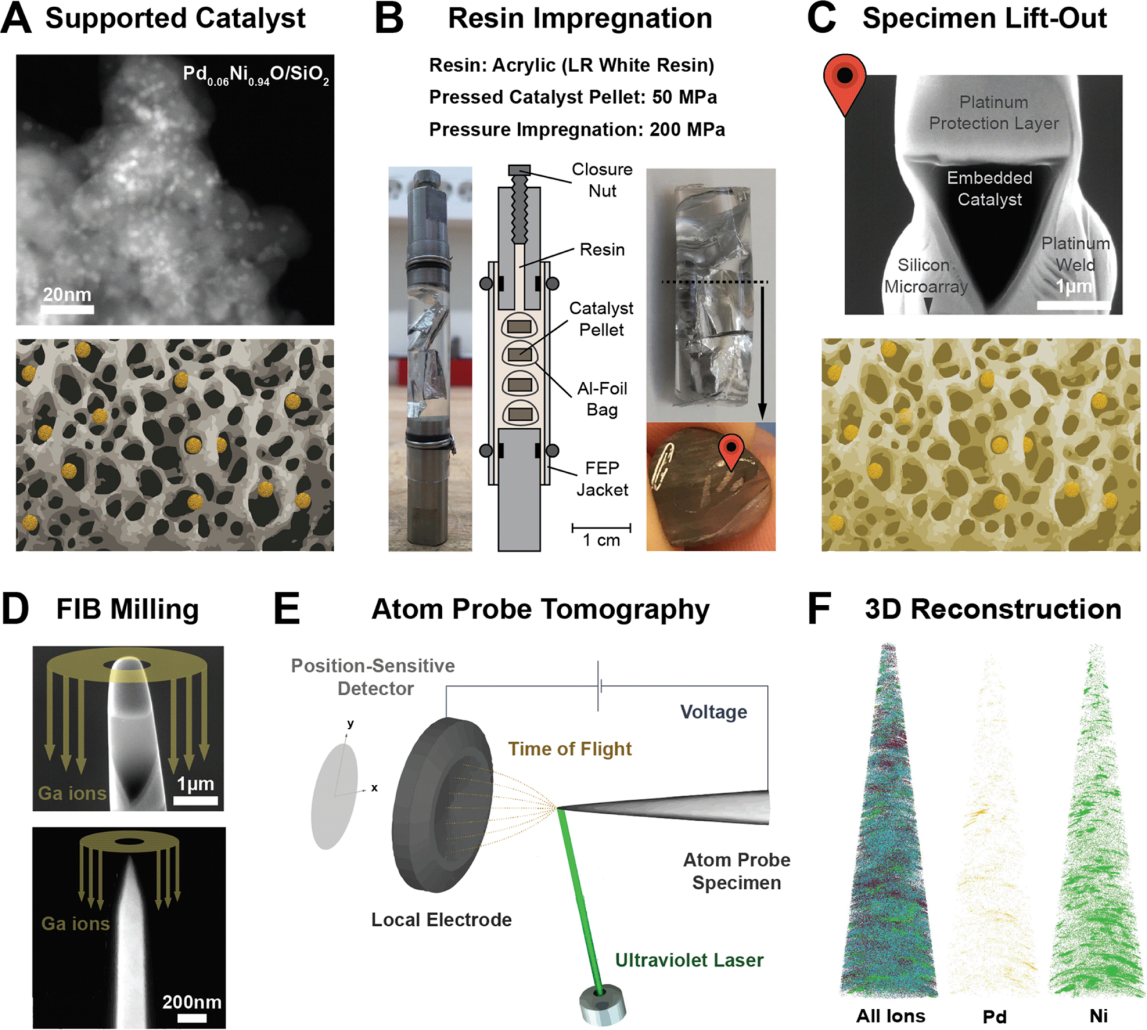
Atom probe tomography (APT) of mesoporous supported catalyst materials. (A) High-angle annular dark-field scanning transmission electron microscopy (HAADF-STEM) image of the Pd_0.06_Ni_0.94_O/SiO_2_ catalyst after calcination (top). Schematic representation of the pristine supported bimetallic catalyst (bottom). (B) High-pressure-assisted resin impregnation approach used to fill the pores of the catalyst. Image of the stub obtained after resin impregnation containing the sample-filled aluminum bags, here dubbed “calzone” (left). Setup used to perform the resin impregnation (middle) and cross-section obtained after cutting and polishing with the indicated lift-out position (red needle) (right). (C) Image of the lifted-out sample welded on top of a silicon microarray tip (top; for details, see Figure S10). Schematic representation of the resin-impregnated supported bimetallic catalyst (bottom). (D) Images displaying the first (top) and second (bottom) annular focused ion beam (FIB) milling steps used to prepare the atom probe specimen. (E) Illustration of the measurement principle of APT. (F) Reconstructed data of the resin-embedded Pd_0.06_Ni_0.94_O/SiO_2_ catalyst obtained from APT. Pixels in reconstruction: yellow (palladium), green (nickel), gray (silica), purple (carbon), and blue (oxygen).

Within this context, atom probe tomography (APT) is a unique analytical tool, as it offers three-dimensional sub-nm spatial resolution with exceptional compositional sensitivity.^18^ This makes the technique appealing for studying compositional heterogeneities for large numbers of small metal nanoparticles, complementary to the sub-100 nm−μm scale probed by STXM.^19^ Typically, for probing samples with APT, a needle-shaped specimen is prepared by focused ion beam (FIB) lift-out and milling procedures.^20^ The obtained specimen is then field evaporated in the atom probe in an ion-by-ion fashion with the help of an applied electric field and laser (or voltage) pulses (Figure 1E).^18^ The thus-generated ions fly toward the position-sensitive detector guided via a local electrode and analyzed by time-of-flight mass spectrometry (TOF-MS). Specimens are then reconstructed via the pulse sequence of the laser, time-of-flight, and positions from the detector. The reconstructed data then provide a 3D snapshot of the specimen, which can be analyzed, for example, by clustering algorithms to obtain the individual nanoparticle structures.

APT has already been applied in the past to unsupported metal (oxide) nanoparticles using different embedding approaches (Table S1). However, to the best of our knowledge, APT was not yet performed to study metal nanoparticles supported on mesoporous oxide materials, typically used in industrial catalyst materials. The main reason for this is the high porosity of these solid catalysts (far beyond 50 vol %). While needed in catalysis to allow for sufficient mass transport, the presence of pores causes issues during (i) specimen preparation because of the lack of mechanical stability of the material during FIB lift-out and milling procedures,^20,21^ (ii) data acquisition where the strong electric fields can cause premature rupture of the specimen,^21−23^ and even if data are acquired, issues arise during (iii) data reconstruction as the pores deform the hemispherical shape of the tip, causing ion trajectory aberrations.^21,23−25^ These challenges limited APT to mostly nonporous samples in the past.^22,26,27^ Efforts in the past decade made it possible to study microporous materials, such as zeolites, by preparing APT tips from microcrystals of at least 1 μm in size.^19,28−33^ However, this approach is not applicable to small crystallite sizes or mesoporous materials. Most recently, a macroporous metal oxide was investigated with a melt infiltration approach using fusible metals. While this approach may enable probing certain porous materials, it cannot be generally applied for supported metal catalysts because of the dissolution of metals in the presence of fusible alloys.^34,35^ Another approach involved the solvatization of nanoparticles and subsequent electrophoretic deposition on top of a presharpened AP tip.^36^ While this strategy enabled the study of commercial carbon-supported catalysts, a limitation of this deposition-based approach is the probed volume. Therefore, improvement via direct AP tip fabrication from self-standing materials would present a facile approach to study larger numbers of nanoparticles.

Here, we adapted a resin impregnation approach inspired by previous studies on both biominerals and proteins^37−40^ and its well-known application for making microtomes of porous samples for TEM analysis, to embed an industrial catalyst material consisting of bimetallic palladium and nickel metal nanoparticles supported on mesoporous silica (with a surface area of 300 m^2^/g and a pore volume of 1.3 cm^3^/g) for subsequent APT preparation and analysis. We showcase how APT can yield insights into large-scale catalyst preparation procedures, such as incipient wetness co-impregnation (co-IWI). These procedures comprise a simple workflow starting with metal precursor impregnation, followed by drying, calcination, and often reduction to obtain the supported metallic catalyst. Practical simplicity, low-waste streams, and its water-based nature make this approach appealing for industrial catalyst preparation.^41^ However, when applying co-IWI to Pd–Ni-based bimetallic catalysts, controlling metal segregation phenomena can become a primary challenge, compromising the catalysts’ performance.^3,10,41^ With the help of APT, nanoscale compositional heterogeneities can be statistically assessed by providing a snapshot of the catalyst material, enabling us to guide the rational design of the catalyst preparation. We envision that this methodology can expand the capabilities of APT toward the characterization of other porous materials such as porous electrodes and membranes.

## Results and Discussion

### Synthesis and Characterization of Bimetallic Pd–Ni Catalysts

The silica-supported Pd–Ni catalyst (Pd_0.06_Ni_0.94_O/SiO_2_) under investigation was chosen to represent a low noble metal content bimetallic catalyst for two intertwined reasons: First, the scarcity of palladium and the associated high price generally favor the addition of Pd in small quantities to most efficiently utilize noble metals. Related to this catalyst design argument, we further aim to demonstrate APT’s capabilities to statistically evaluate the spatial distribution of trace components.

The catalyst material was synthesized by co-IWI (Figure 1A). After impregnation and drying, the catalyst material was calcined under 1 vol % NO/N_2_. This resulted in the formation of partially segregated NiO and PdO, based on XRD, XAS, and TPR characterization (Figures S1–S3). This was further corroborated by STEM-EDX, which showed the presence of nickel- and palladium-rich areas (Figure S4). In accordance with the low average palladium content of 1 wt %, predominately nickel-rich areas were found. The observed compositional heterogeneities motivated our efforts in assessing inter- and intraparticle metal segregation at the sub-nanoscale by APT.

### Embedding Approach and Atom Probe Specimen Preparation

After calcination, the supported catalyst had a pore volume of around 1.3 cm^3^/g, which corresponds to a porosity of ∼70 vol % with an average pore diameter of ∼12 nm (Figure S5). To prepare an almost void-free specimen for APT, different pore-filling materials were tested, initially including silica from water glass (see SI for details). From the perspective of keeping a rather homogeneous field evaporative behavior, silica is preferred, as no additional material is added and evaporation fields of the elements are rather similar. However, due to the weak mechanical stability during lift-out FIB milling procedures, premature tip failure occurred or deformed tips were yielded, which were not compatible with APT analysis (Figure S6). To improve the mechanical strength of the tip, we turned to resin materials, specifically acrylic resins (Figure S7). However, due to polarity differences between resin matrix and silica, pore filling required external pressure to be applied, for which we designed a high-pressure “calzone”-type approach (Figure 1B). A hydrostatic resin pressure of 200 MPa was chosen to facilitate pore filling, while avoiding irreversible deformation of the supported catalyst material.^42,43^ For this, a pellet of the catalyst material was pressed and placed into an aluminum foil, envelope-type bag (calzone). Subsequently, the calzone was placed in a small sample assembly consisting of two steel pistons and a fluorinated ethylene propylene (FEP) jacket. The catalyst-filled bag was submerged in resin prior to the application of pressure to allow for pore filling of the silica. The obtained resin-impregnated assembly was further hardened and cross-sectioned. Successful APT tips for analysis were obtained when using a low-viscosity acrylic resin (8 mPas). After cross-sectioning the stubs, SEM-EDX was performed to inspect the remaining porosity (Figure S8) and the degree of pore filling (Figure S9). Both SEM images and EDX maps indicated the presence of carbon within and surrounding the silica spheres, suggesting resin penetration. Despite the high pressures applied during resin impregnation, we have noted that there is no significant sign of nanoparticle migration or redistribution, which was further corroborated by EELS (Figure S12). To allow for capturing large numbers of nanoparticles within the AP tip, EDX mapping was performed for selecting a lift-out position containing a high concentration of nickel atoms. The sample was then lifted out with the FIB (Figure 1C) and cut into four wedge-shaped prisms, and each prism was milled to obtain the final atom probe specimen (Figure 1D) (for more details see Figure S10). Subsequently, the APT analysis was performed (Figure 1E) and the tip was reconstructed (Figure 1F).

### Atom Probe Tomography of the Embedded Catalyst Material

The APT data reconstruction comprises a volume of 3 × 10^5^ nm^3^, showing the distribution of Si, O, C, Ni, and Pd within the specimen (Figure 1F, Figure S13, and Movie S1). While heterogeneous multicomponent specimens often lack data accuracy due to evaporation field differences between constituents,^44,45^ distinguishable high concentration (HC) areas can be spotted for Pd and Ni. This further illustrates that although the average bulk concentration of Pd is around 0.05 at. %, not only can Pd be detected but also its spatial distribution can be assessed. When inspecting these HC areas, axial distortions are visible. Found commonly for nanoparticle-containing samples in APT, this artifact has its origin in local evaporation field differences.^46,47^ However, although distorted, the limited overlap between areas is spotted, allowing for separation between the individual nanoparticles. Nevertheless, inspecting the sample via the reconstruction is not per se intuitive and lacks quantitative information. Hence, advanced data processing approaches are necessary to inspect the data in more detail to extract the individual nanoparticle structures.

**Figure 2:**
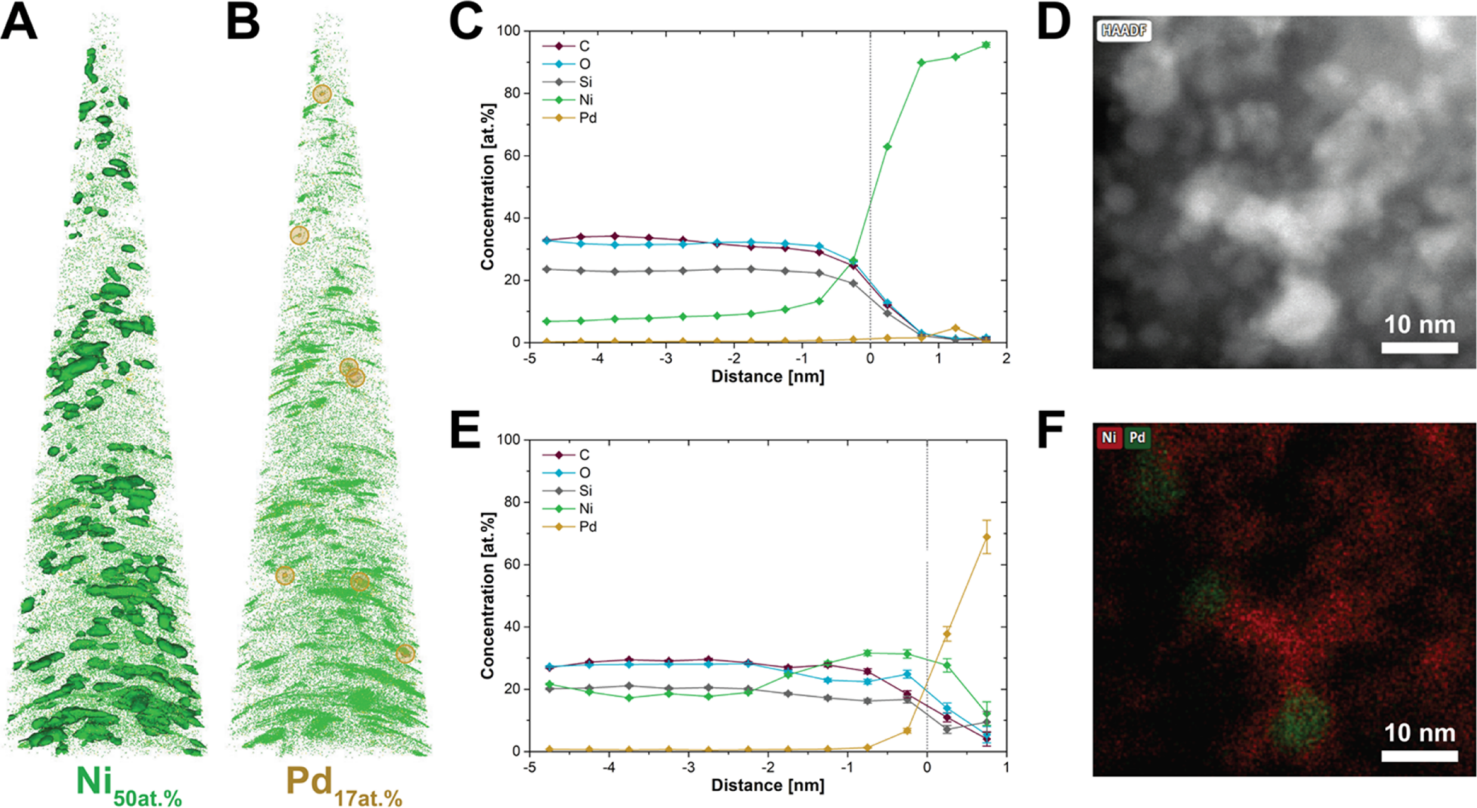
Nanoscale distribution of Ni and Pd in the resin-embedded Pd_0.06_Ni_0.94_O/SiO_2_ catalyst visualized by atom probe tomography (APT). (A, B) Truncated cone-shaped data reconstruction of Pd_0.06_Ni_0.94_O/SiO_2_ with *h*: 148 nm, *r*: 5 nm, *R*: 41 nm, *V*: 3 × 10^5^ nm^3^. Isoconcentration surface (ICS) analysis for (A) Ni (50 atom %) and (B) Pd (17 atom %) displayed the high-concentration areas for the elements. (C, E) Corresponding proximity histograms of (C) 50 atom % Ni ICSs and (E) 17 atom % Pd ICSs displayed in (A) and (B), respectively. Positive and negative distances reflect the inside and outside of the ICS, respectively. (D, F) Scanning transmission electron microscopy coupled with energy-dispersive X-ray spectroscopy (STEM-EDX) imaging of Pd_0.06_Ni_0.94_O/SiO_2_ (Pd in green and Ni in red).

Isoconcentration surface (ICS) analysis allows inspection of compositional heterogeneities within a sample and the description of the average concentration profiles of elements within those ICSs as a function of distance from the delimiting surface. In ICS analysis, a 3D grid in concentration space is created by connecting voxels (3D pixels) of equal concentration. Concentration thresholds were arbitrarily chosen to extract the HC areas. Obtained ICSs for both Ni and Pd showcase the distribution, size, and shape of the HC areas (Figure 2A and 2B). While many HC areas were found for Ni, only seven areas for Pd were found in the APT data. This is consistent with STEM-EDX results, which show the presence of Ni- and Pd-rich areas (Figure 2D and 2F). Although some distorted nanoparticle-shaped ICSs could be isolated for Ni, part of them seems to congeal nanoparticles in proximity. To further inspect these HC areas, proxigrams (proximity histograms) were derived (Figure 2C and 2E). Proxigrams display the average atomic concentration of the elements as a function of the distance from the ICS, inside (positive numbers) and outside (negative numbers) of the ICSs. The Ni proxigram (Figure 2C) illustrates that Pd and Ni concentrations are correlated, while matrix elements (C, Si, and O) are anticorrelated with Ni. The Pd proxigram showed above-average concentrations of Ni close to the ICS and 10 at. % Ni even inside the ICS (Figure 2E). Although the statistical relevance of the proxigram of Pd is limited by the small number of Pd-ICSs, this suggests that the Pd-rich areas of the sample are generally surrounded by Ni-rich shells.^48^

The observed C and Si anticorrelation with Ni and Pd was expected, given that Si is found in the support and C in the resin surrounding the catalyst. On the other hand, the low (apparent) O concentration in the ICS was in contrast with the presence of NiO and PdO revealed in both XAS and XRD results of the catalyst material (Figures S1 and S2). Interestingly, mass spectrometry (MS) patterns obtained during APT analysis did not detect oxygen-containing mass fragments (Figure S14). When the mass fragments of the specimen are compared with reference materials, a resemblance with the pattern of bulk metallic nickel is found. While bulk nickel oxide shows peaks for NiO_*x*_^+^ species, the absence of these peaks suggests that metallic nickel is present according to APT analysis. Therefore, two main hypotheses would describe the aforementioned observations: (i) metal oxide reduction occurred during resin impregnation or FIB milling or (ii) significant oxygen loss took place during APT. According to TPR analysis, the reduction of nickel oxide generally happens at high temperatures in reducing atmospheres (Figure S3). Given that the base temperature in the analysis is around 50 K and that laser pulsing only increases temperatures to 100–300 K, we do not expect this to happen under analysis conditions.^49,50^ Furthermore, during resin impregnation, temperatures only reach around 330 K, which we expect to be insufficient to reduce NiO based on TPR results (Figure S3). Nevertheless, reduction during resin impregnation and FIB milling was further investigated. Selected-area electron diffraction (SAED) performed on a FIB-milled lamella fabricated from the resin-impregnated catalyst materials confirmed the oxidic nature of the nanoparticles found by XRD, XAS, and TPR (Figure S11). STEM-EELS furthermore showed the absence of large reduced nickel nanoparticles (Figure S12). While the absence of oxygen in APT has to be further investigated, we suspect that the nanosized nature of the material, evaporation field differences between the materials, and high laser powers (100 pJ) might exacerbate the loss of oxygen found for many oxidic materials.^51−53^ Loss of oxygen might arise from evaporation as neutral species or molecular ions in conjunction with matrix atoms, resulting in a loss of NiO_*x*_^+^ species. It would be interesting to compare APT data from an APT system with higher laser energy, but this is beyond our capabilities.

**Figure 3:**
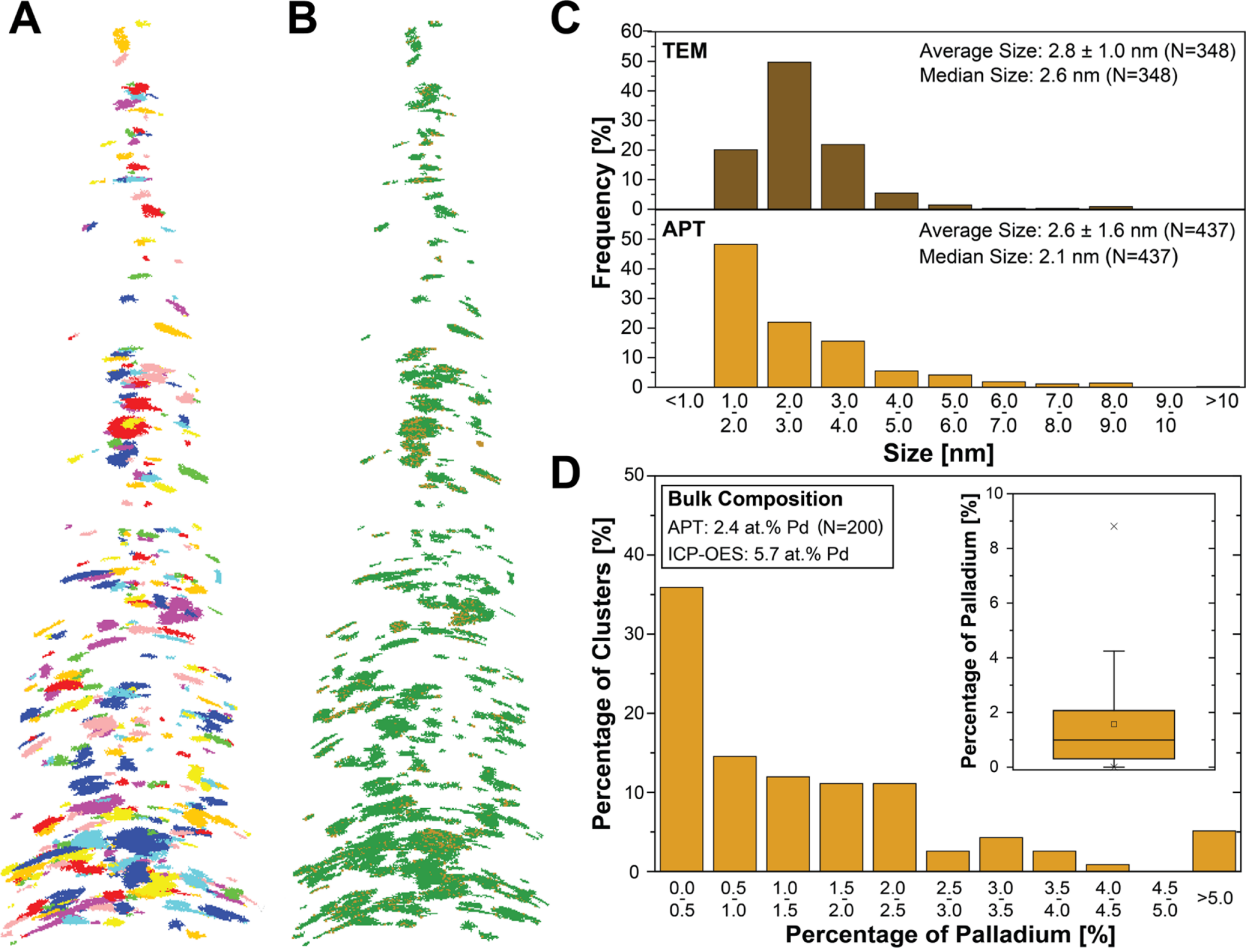
Size, location, and single-particle composition of Pd–Ni clusters in reconstructed Pd_0.06_Ni_0.94_O/SiO_2_. (A) Results of the cluster analysis performed on the reconstructed resin-embedded Pd_0.06_Ni_0.94_O/SiO_2_ tip using the maximum separation method (see SI for details, *d*_max_: 0.3 nm, *N*_min_: 16, *L*, *E*: 0.8*d*_max_), where clusters are depicted in different colors to be identified. (B) Palladium (ocher) and nickel (green) atoms within the identified clusters show the heterogeneity in Pd distribution. (C) Particle size histograms obtained from high-angle annular dark field-scanning transmission electron microscopy (HAADF-STEM) imaging (top) and APT cluster analysis (bottom). The cluster size was calculated based on the number of atoms detected, assuming a spherical oxidic nanoparticle model and a detector efficiency of 33% (eq S1). (D) Histogram showing the distribution of single-cluster Pd concentration, obtained from cluster analysis (for clusters with >300 atoms ≙ 1.85 nm), as *N*_Pd,cluster_/(*N*_Ni,cluster_ + *N*_Pd,cluster_). Inset: corresponding box and whisker plot. The average Pd at. % in Ni–Pd nanoparticles was obtained from APT based on the total number of atoms in all clusters: *N*_Pd,total_/(*N*_Ni,total_ + *N*_Pd,total_) and from elemental analysis (ICP-OES, inductively coupled plasma-optical emission spectrometry).

While ICS analysis enables the isolation of HC areas and visualizes the average proximity between metals, separation between individual nanoparticles and obtained spatial information is limited. One-dimensional concentration profiles (1DCP, Figure S15) display the presence of spatial heterogeneities, however lack quantification of compositional heterogeneities. Therefore, cluster analysis (Figure 3) was performed based on the maximum separation method, where clustering of solute atoms was done by an iterative approach. The approach is based on two independent variables: the maximum distance between solute atoms *d*_max_ and the minimum number of solute atoms *N*_min_. As these parameters strongly influence the clustering result, a sensitivity analysis was performed to optimize clustering (Figures S16–S18). For compositional analysis, ranging of mass fragments was further optimized to obtain Pd–Ni metal ratios (see SI for details and Figure S19).

Within the specimen, 437 clusters were identified (Figure 3A and Movie S2 (fourth from the left)), with the corresponding distribution of Pd and Ni (Figure 3B). Size analysis obtained from cluster analysis yielded a size distribution for the clusters of 2.6 ± 1.6 nm (Figure 3C). On the other hand, HAADF-STEM-based size analysis found a comparably narrow size distribution of 2.8 ± 1.0 nm. We ascribe the broader size distribution from APT to the (i) detection of smaller clusters in APT as well as (ii) separation issues using cluster analysis (inability to separate close nanoparticles and separation of nanoparticles into sub-nanoparticles). Furthermore, artificial small nanoparticles might originate from inaccuracies in the 3D reconstruction related to trajectory aberrations. This showcases how our understanding of the catalysts’ size distribution can be extended by APT. While determining the size histogram remains an intrinsic limitation of the clustering method, the correlative analysis approach might help to resolve and/or limit this issue.^46,47^

However, while size information can be obtained by various techniques, APT additionally provides compositional information for each cluster. Cluster analysis captured around 59 at. % of the metal atoms (labeled as metals) in the full APT data set (44 at. % of Pd and 59 at. % of Ni) and incorporated around 2 at. % of the matrix atoms (Si and C). For a nonaccurate relaxed parameter choice (*d*_max_ = 0.5 nm) only around 72 at. % of metal atoms were captured (60 at. % of Pd and 73 at. % of Ni), alongside incorporating around 12 at. % of matrix atoms (Figures S17 and S20). These losses can be attributed to a multitude of factors such as (i) signal-to-noise issues (more pronounced for Pd), (ii) the presence of smaller clusters (below the minimum cluster size *N*_min_), (iii) clustering issues (e.g., trajectory aberrations that artificially increase the distance between atoms), as well as (iv) spectral interferences (e.g., ^60^Ni^+^ and ^28^Si^16^O_2_^+^). To illustrate the influence of spectral interferences, we limited the quantification of Ni to ^58^Ni^+^. An increased number of captured Ni atoms by cluster analysis was found with values of 75 at. % and 89 at. % for *d*_max_ values of 0.3 and 0.5 nm, respectively. We suspect that most of the remaining Ni atoms which were not captured by cluster analysis were either subject to trajectory aberrations or represent ion-exchanged single-atom moieties on the silica surface.

When the distribution of Pd and Ni within the clusters was inspected (Figure 3B), HC areas for both elements were spotted, corroborating the suspected heterogeneities within the specimen. The composition histogram as well as the corresponding box and whisker plot further display the distribution of Pd concentration for the individual clusters (Figure 3D). Here, a large fraction of clusters (around 35%) containing <0.5 at. % Pd was observed. Furthermore, when looking at the fraction of palladium atoms per total metal atoms within the clusters, a large discrepancy is found between APT with [Pd] = 2.4 at. % and elemental analysis with [Pd] = 5.7 at. %. While 2.4 at. % Pd includes only the fraction of larger clusters (>300 atoms, > 1.85 nm), the same general trend with an average composition of 2.9 at. % Pd was found for the fraction of small clusters (<300 atoms, <1.85 nm). These observations are consistent with the fact that we selected a nickel-rich part of the sample for lift-out and APT specimen preparation. The results are also in line with STEM-EDX analysis, which showed predominately nickel-rich nanoparticles with small numbers of palladium-rich nanoparticles (Figure S4). This further decreases the probability of finding Pd-rich (>50 at. %) nanoparticles given that the number of atoms scales with diameter as follows: *N*_M_ ∝ *d*_NP_^3^. While the results allow us to statistically evaluate the compositional heterogeneity on the nanoscale, given that 200 nanoparticles (with >300 atoms) were probed, the probed volume is still an intrinsic limitation of APT, asking for complementary analysis approaches (e.g., in combination with STXM). Additionally, as varying Pd concentrations were found for the clusters, we inspected the Pd concentration as a function of cluster size and found no correlation (Figure S21).

**Figure 4:**
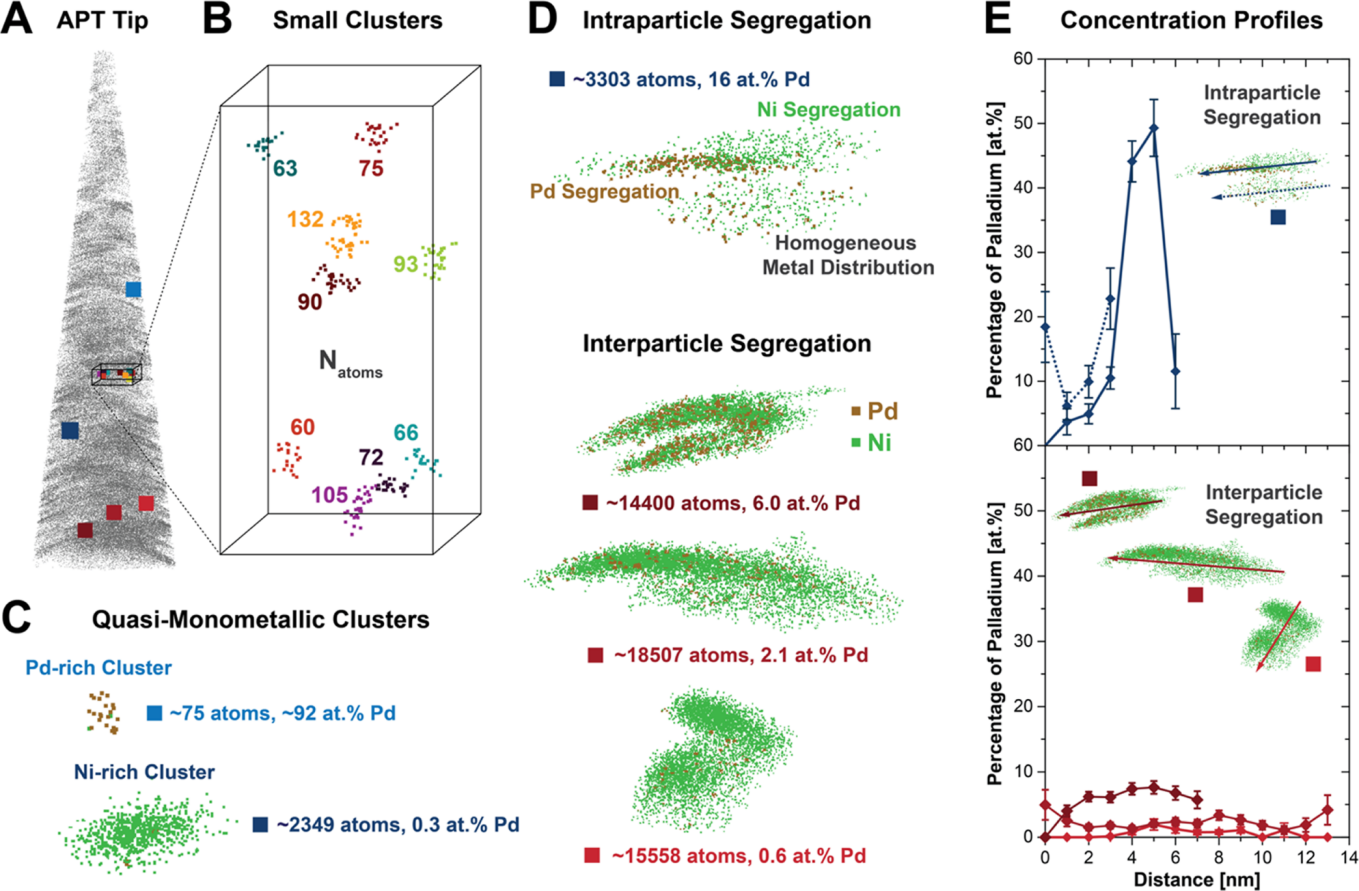
Showcasing heterogeneity at the nanoscale in Pd_0.06_Ni_0.94_O/SiO_2_: small, big, and segregated clusters. (A) Reconstructed tip overlaid with the position of the individual cluster described in B, C, and D. Clusters appear distorted due to the well-known local magnification effect.^46,47^ (B) Representative small Pd–Ni clusters of less than 150 atoms (numbers in panel: nickel and palladium atoms per cluster). (C) Segregated clusters comprised mostly palladium or nickel. (D) Intraparticle and interparticle segregation illustrated by the elemental distribution of palladium and nickel within relatively large clusters (4–9 nm). (E) One-dimensional concentration profiles (1DCP): quantitative assessment of compositional heterogeneities within the corresponding clusters in D. Comparison of 1DCPs for (i) heterogeneous (solid line) and homogeneous (dashed line) subsections, within clusters prone to intraparticle segregation (top panel) and (ii) homogeneous clusters of different average palladium content illustrating interparticle segregation over intraparticle segregation (bottom panel; for analysis details see Figure S22).

To illustrate the clusters used for the aforementioned statistical analysis, we compiled different groups of cluster types representing the heterogeneities within the sample (Figure 4). Although generally significant X–Y distortions were found for most clusters, this does not significantly affect compositional analysis. Distortions arise from the well-known local magnification effect, causing the reconstruction to yield nanoparticles deformed in one dimension.^46,47^ Correlative analysis approaches using STEM can be further applied to correct for this deformation of nanoparticle shapes.^46^

Provided that APT analysis yielded a larger fraction of small clusters compared to HAADF-STEM (Figure 3C), we illustrate some of these small clusters within the tip (Figure 4A and 4B). While 20–44 metal atoms were detected, cluster sizes were assumed to be 60–132 atoms (detector efficiency of 33%), corresponding to sizes of around 1.3–1.7 nm (eq S1). As clusters of this size are at the limit of what could be detected by HAADF-STEM, part of this fraction of small clusters was excluded from the HAADF-STEM-based histogram. However, although some of these clusters showed distorted shapes or partial overlap between clusters, generally well-separated clusters were identified.

Furthermore, almost monometallic clusters with a minor solubility for the other metal were discovered (Figure 4C). Although the tip represents a nickel-rich area of the sample, the presence of a single small palladium-rich nanoparticle (∼1.5 nm, ∼92 at. % Pd) confirms the presence of Pd-rich nanoparticles, in accordance with STEM-EDX (Figure S4). The nickel-rich nanoparticle, on the other hand, represents one of many nickel-rich nanoparticles as derived from the compositional histogram (Figure 3D). The presence of these nanoparticles (of different compositions) showcases that the sample was subject to interparticle metal segregation (between nanoparticles).

To inspect metal segregation phenomena in more detail, we investigated the metal distribution within clusters as illustrated for four clusters of different Pd concentrations (Figure 4D). With the help of 1DCPs (Figure 4E) these qualitative visual inspections can be further quantified, allowing us to assess the degree of compositional heterogeneity within the nanoparticles. For this, a 2D cross-section enclosing the entire cluster is analyzed as 1DCP along its longest axis (Figure S22).

When inspecting clusters, a generally homogeneous distribution of palladium and nickel within the cluster is observed for most clusters, as represented by the bottom three clusters. This is further confirmed by the 1DCPs which show rather limited deviations within the concentration profile. This indicates limited segregation within the nanoparticle (intraparticle), while significantly different concentrations between clusters corroborate that metal segregation is predominately occurring between nanoparticles (interparticle).

On the other hand, the displayed sections of the top cluster showed significant compositional differences within subsections of the cluster, ranging from nickel-rich to homogeneous to palladium-rich. Although this suggests the presence of intraparticle metal segregation, only very limited sections were found throughout the tip, complicating a statistical assessment of this phenomenon. Furthermore, we stress that interpreting the shapes of these clusters is not trivial. Because the sample is the primary optic in APT, intrinsic differences in field evaporative behavior can affect the spatial accuracy of data.^18,24,51^ This complicates the assessment, especially for large clusters, as one cluster can represent multiple congealed nanoparticles or just a single distorted nanoparticle. Nevertheless, 1DCP analysis of the top clusters was conducted through the heterogeneous and homogeneous subsection of the cluster. Concentration profiles strongly indicate the difference in compositional heterogeneity given that spread between compositions is between 6–23 at. % and 0–50 at. % for the homogeneous and heterogeneous sections, respectively.

These findings underline that prior synthesis steps predominantly yielded interparticle metal segregation. We hypothesize that interparticle segregation was mainly caused by the combination of (i) spatial heterogeneities in the metal precursor distribution (from impregnation and drying) and (ii) deviations in metal oxide formation (during calcination). Therefore, we suspect that maintaining a homogeneous distribution of the metals during impregnation and drying is insufficient for preventing compositional heterogeneities (including uniform size and spatial distribution of the nanoparticles). We suggest that, additionally, control over metal precursor decomposition (e.g., via the calcination conditions) is necessary to achieve uniform nucleation and growth dynamics across the support material. Although further research is needed to demystify the origin of metal segregation phenomena in these catalyst materials, we were able to demonstrate how the APT can aid this process. By probing compositional heterogeneities on the nanoscale, we shed light on the structural implications of synthesis procedures necessary for the rational design of uniform catalyst materials.

## Conclusions

We have demonstrated a novel sample preparation approach for studying mesoporous materials, containing bimetallic nanoparticles, with atom probe tomography, based on acrylic resin embedding. Our results showcase that APT deepens our fundamental understanding of supported bimetallic nanoparticles, active in CO_2_ hydrogenation, by providing intra- and interparticle compositional information for larger numbers of metal nanoparticles, which are largely inaccessible by other analytical approaches. In essence, the catalyst material shows significant metal segregation, which is reflected in the separation into nickel- and palladium-rich nanoparticles. While immiscibility between oxide phases due to crystallographic differences causes intrinsic segregation, APT illustrated that the presence of palladium oxide within nickel oxide is occurring, implying that some degree of a solid solution has been formed. With the help of cluster analysis, a total of 437 metal nanoclusters were captured, with sizes of 2.6 ± 1.6 nm and an interquartile compositional range of 0.3–2.1 at. % Pd. While the intraparticle segregation of Ni and Pd was observed only in a few cases, interparticle metal segregation was predominant. This suggests that controlling the metal precursor distribution during impregnation and drying as well as their decomposition upon calcination is crucial for preventing compositional heterogeneities. Our results underline that APT is capable of quantitatively assessing compositional heterogeneities down to the scale of very small metal nanoparticles, thereby further illustrating that it is a powerful tool for the characterization of porous anisotropic functional nanomaterials. We envision that the novel approach developed allows us to expand the unique features of the APT technique toward various disciplines of material science, including but not limited to the characterization of catalysts, fuel cells, and batteries.

## Supporting Information Available

Movie S1: 3D reconstruction from left to right of all ions, Si, C, O, Pd, and Ni (MP4)
Movie S2: Cluster analysis for *d*_max_ values from left to right of 0.10, 0.15, 0.20, 0.30, 0.40, and 0.50 nm (MP4)
Experimental section (catalyst synthesis, catalyst characterization, resin impregnation, and atom probe tomography), literature overview of embedding strategies, ICP-OES, XRD, XAS, TPR, CO_2_ hydrogenation, STEM-EDX, and N_2_ physisorption analysis of the catalyst materials, FIB lift-out, and milling procedures using silica and resin-embedding approaches including cross-section analysis with SEM and STEM-EDX, EELS and SAED analysis of the FIB-milled lamellas, APT 3D reconstructions, TOF-MS analysis of the catalyst and reference materials, 1DCP analysis, sensitivity analysis for clustering (PDF)
