# Supplementary material for: Elucidating the Structure and Composition of Individual Bimetallic Nanoparticles in Supported Catalysts by Atom Probe Tomography
Source: J Am Chem Soc. 2023 Jul 25;145(31):17299–308. doi: 10.1021/jacs.3c04474 (PMC10416302; doi:10.1021/jacs.3c04474)
Supplement: Supplementary file 3 — ja3c04474_si_003.pdf [file ja3c04474_si_003.pdf]

Supporting Information

Elucidating the structure and composition of individual bimetallic nanoparticles in supported catalysts by atom probe tomography

Florian Zand[1], Suzanne J. T. Hangx[2], Christopher J. Spiers[2], Peter J. van den Brink[3], James Burns[4], Matthew G. Boebinger[4], Jonathan D. Poplawsky[4,*], Matteo Monai[1,*], and Bert M. Weckhuysen[1,*]

[1]Inorganic Chemistry and Catalysis Group, Institute for Sustainable and Circular Chemistry and Debye Institute for Nanomaterials Science, Utrecht University, Utrecht 3584 CG, The Netherlands
[2]High Pressure and Temperature Laboratory, Utrecht University, Utrecht 3584 CB, The Netherlands
[3]Shell Global Solutions, Amsterdam, 1031 HW, The Netherlands
[4]Center for Nanophase Materials Sciences, Oak Ridge National Laboratory, Oak Ridge 37831, Tennessee, United States
**E-mail: poplawskyjd@ornl.gov, m.monai@uu.nl, b.m.weckhuysen@uu.nl*

Experimental Section

Catalyst materials synthesis

Silica-supported Ni, Pd, and Pd-Ni catalysts were prepared by incipient wetness impregnation (IWI). 2 g support powder (CARiACT Q-10, Fuji Silysia Chemical, V_{Pores}:1.0 cm^3/g) was pre-dried under stirring at 230 °C for 2 h under dynamic vacuum (2-3 mbar). Subsequently, the powder was cooled to room temperature and impregnated drop-by-drop with metal precursor solution under static vacuum conditions and rigorous stirring. For a metal precursor solution, nickel nitrate hexahydrate (Sigma Aldrich, 99.999 %) and tetraammine palladium nitrate (Sigma Aldrich, 10 wt.% in water) were dissolved in deionized water. The impregnated catalyst materials were dried under dynamic vacuum conditions while stirring for at least two days at room temperature. The catalyst material was calcined in a fluidized bed reactor. The calcination procedure was performed in 1 vol.% NO/N_2 at a Gas Hourly Space Velocity (GHSV) of 13,000 h^{-1}, a heating rate (r_{Heat}) of 1 °C/min to a maximum temperature (T_{max}) of 450°C (Ni: 350°C), and a duration at maximum temperature (t_{max}) of 2 h.

Catalyst materials characterization

X-ray Diffraction (XRD) was performed on a Bruker AXS D2 Phaser with Co source (Kα, λ = 1.790 Å). The calcined powder catalyst materials were measured between two-theta angles of 37-57° in increments of 0.05° for 6 s each. XRD patterns were base-line subtracted.

X-ray Absorption Spectroscopy (XAS) was conducted at the SuperXAS beamline of the Swiss Light Source (SLS, Villigen, Switzerland) using a Si (111) monochromator. Self-supporting wafers of the calcined powder catalyst materials were pressed with a tablet press and subsequently measured in transmission mode. The edge positions were calibrated by nickel and palladium reference foils. Spectra were normalized pre- and post-edge with a first- and third-order polynomial, respectively.

Temperature-Programmed Reduction (TPR) analyses were performed on a Micromeritics Auto Chem II 2920 instrument equipped with a Thermal Conductivity Detector (TCD). For the analysis, the sieved calcined powder catalysts (50 mg, <38 µm) were first pre-dried in Ar (50 mL/min, T_{max}:120 °C, r_{Heat}:10 °C/min, t_{max}: 15 min) and subsequently reduced in 5 vol.% H_2/Ar (15 mL/min, T_{max}:900 °C, r_{Heat}: 5 °C/min). The TPR profiles are displayed as unmodified data.

Scanning Transmission Electron Microscopy coupled with High-Angle Annular Dark-Field (HAADF-STEM) detection and Scanning Transmission Electron Microscopy coupled with Energy-Dispersive X-ray (STEM-EDX) spectroscopy imaging were both performed using an FEI Talos F200X Talos instrument equipped with four silicon-drift Super-X detectors in the column. The calcined powder catalyst materials were brought onto Lacey Carbon Film Cu grids via dip coating. Due to the silica-supported nature of the metal nanoparticles, charging and drifting difficulties occurred. As the metal nanoparticles were found to be mobile in the presence of the electron probe, EDX collection was continued until no substantial changes occurred to the region of interest. Particle size histograms were obtained by manually measuring at least 300 metal nanoparticles from images of both different regions as well as magnifications.

Furthermore, both STEM and Transmission Electron Microscopy (TEM) were conducted on Focus Ion Beam (FIB) milled lamella lift-outs of the resin-impregnated stub of the Pd-Ni catalyst material (**Figure S11A**). All of these experiments were carried out on a FEI Titan (60-300kV) aberration-corrected S/TEM instrument operating at 300kV for both the TEM and STEM experiments. Selected Area Electron Diffraction (SAED) patterns seen in **Figure S11B-S11C** were taken with a Gatan OneView camera. Additionally Scanning Transmission Electron Microscopy coupled with Electron Energy Loss Spectroscopy (STEM-EELS) was performed on a Gatan Quantum EEL spectrometer at a dispersion level of 0.3 eV/channel and exposure of 0.1 s (**Figure S12**). N_2 physisorption measurements were conducted on a Micromeritics Tristar 3000 Surface Area Analyzer at -196 °C. Prior to the measurements, the powder catalyst materials were degassed under vacuum at 230 °C overnight.

Resin impregnation

Cold-pressing of ~100 mg of the powder catalyst material was done at 50MPa applied stress, in a 5 mm diameter die (~30 min). The obtained catalyst pellet was then transferred into an envelope-shaped aluminum bag. The bag was positioned inside the impregnation holder and the holder was filled with resin (acrylic resin: LR White Resin (Agar Scientific), **Figure S7**). After tightly closing the assembly, it was placed in a pressure vessel, with water as the confining medium. The pressure vessel was brought to 200 MPa hydrostatic pressure and 60°C, to ensure that the low-viscosity resin could penetrate the catalyst pellets. After 48 h, the assembly was removed from the pressure vessel, and the hardened resin with catalyst-filled bags were cut free from the Fluorinated Ethylene Propylene (FEP) jacket. The resin was left to cure for another 48 h in an oven at 50°C. The obtained resin-impregnated stubs were subsequently cut with a diamond saw to reveal the cross-section of the resin-impregnated catalyst sliver. The obtained cross sections were further polished with 4000-grade sanding paper and checked with Scanning Electron Microscopy coupled with Energy-Dispersive X-ray (SEM-EDX) spectroscopy (**Figures S8-S9**). Prior to these measurements, the obtained cross sections were sputter-coated with a 12 nm thick Au layer. SEM-EDX was performed on an FEI Helios Nanolab G3 instrument operated at 2-5 kV (for imaging) and 10-15 keV (for elemental mapping).

Atom probe tomography

Standard lift-out and annular FIB milling procedures were done using an FEI Nova 200 Dual-Beam FIB-SEM instrument.[1] The lift-out positions were identified by SEM-EDX (**Figure S9**). An overview of the procedure applied is depicted in **Figure S10**.
Atom Probe Tomography (APT) was conducted using a CAMECA LEAP 4000XR in laser mode. APT experiments were performed at a 50K base temperature, a 200 pJ laser energy, a 125 kHz pulse repetition rate, and a 0.1% detection rate. The reference APT experimental (Ni and NiO) can be found elsewhere.[2] The reconstruction and data processing was done using CAMECA's IVAS 3.8 software package using the tip profile reconstruction method.

Literature overview

Table S1: Compilation of approaches used for sample preparation to analyze nanoparticles with atom probe tomography (APT)

Analysis Approach	*Literature*
sputter coating	3-5
electron-beam assisted deposition	6
atomic layer deposition	7
melt infiltration	8
aggregated nanoparticles	9
de-supporting of nanoparticles	10

Catalyst Characterization

Table S2: Overview of the studied catalyst materials.

Sample Code	*Weight Loading by ICP-OES [wt.%]*	*Pd:Ni-ratio by ICP-OES [wt.%/at.%]*
$Pd_{0.06}Ni_{0.94}O/SiO_2$	9.9	10:90/6:94
$Pd_{0.15}Ni_{0.85}O/SiO_2$	10.1	24:76/15:85
NiO/SiO_2	10.2	0:100/0:100
PdO/SiO_2	3.5	100:0/100:0

As can be noted from the XRD pattern (**Figure S1**), both reflexes at two-theta angles around 43.3° and 50.5° were observed, corresponding to the 111 and 200 planes of cubic (PDF 00-047-1049) or rhombohedral NiO (PDF 04-011-2340), respectively. These two reflexes were also observed for NiO/SiO_2 and $Pd_{0.15}Ni_{0.85}O/SiO_2$. Additionally, a reflex around 39.6° was found for $Pd_{0.15}Ni_{0.85}O/SiO_2$ corresponding to both 002 and 101 planes of tetragonal PdO (PDF 00-041-1107). While only a slight shoulder is visible for $Pd_{0.06}Ni_{0.94}O/SiO_2$, the XRD hints towards the presence of PdO. The presence of this separate reflex suggests that both NiO and PdO might present (partially) separated phases as a phase-shifted single reflex is expected for miscible oxides according to Vegard's law.[11, 12] While the detection limitations of XRD, do not allow to assess the degree of miscibility (tiny shifts of around 0.1° would be expected), also XAS suggests at least partial phase separation (**Figure S2**). When comparing the nano-sized PdO/SiO_2, NiO/SiO_2 $Pd_{0.06}Ni_{0.94}O/SiO_2$ with bulk references, at both Ni and Pd edge, two observations can be made. First, both palladium and nickel in $Pd_{0.06}Ni_{0.94}O/SiO_2$ seem to be present as oxides as suggested by XRD. Secondly, no significant changes in the extended X-ray absorption fine structure (EXAFS) part of the spectrum can be observed between the nano-sized oxides. This suggests that limited amounts of mixed oxides are present within the sample, given EXAFSs sensitivity towards the average local environment of the absorber. Furthermore, also the TPR (**Figure S3**) hints towards the heterogeneity of the sample with both reduction processes at 100-150°C, 180-370°C, and 370-500°C. While generally lower reduction temperatures are observed for the bimetallic catalyst in line with literature,[13, 14] these changes might be ascribed to the presence of metallic palladium which assists in hydrogen dissociation and spillover.[15, 16] Still heterogeneities within the pattern suggest that partial segregation between the metal oxides causes part of the nickel to experience less assistance with reduction.

In addition to these average properties, STEM-EDX imaging reveals the spatial distribution of the elements showcasing the presence of significantly different regions. While most of the catalyst sample is best represented by the top row of images (**Figure S4D**-**S4L**), also regions containing Pd-rich nanoparticles were observed (**Figure S4A**-**S4C**). In general, a trend is observed that Pd-rich nanoparticles grow larger than their Ni-rich counterparts.

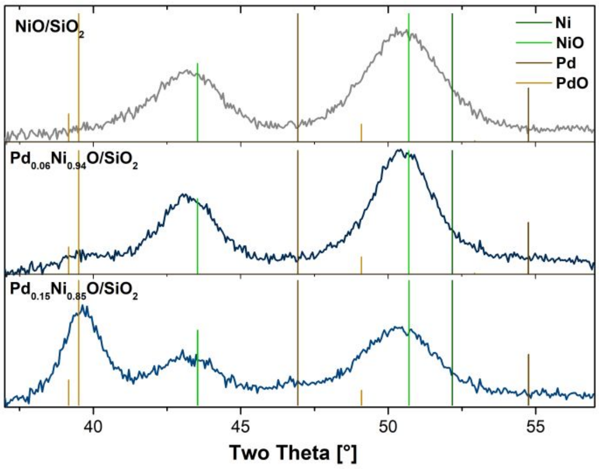

Figure S1: *Ex situ* X-ray diffraction (XRD) pattern from bottom to top: $Pd_{0.15}Ni_{0.85}O/SiO_2$, $Pd_{0.06}Ni_{0.94}O/SiO_2$, and NiO/SiO_2. Reference pattern sourced from ICDD database. Reference patterns: Ni (dark green; cubic PDF 00-004-0850), NiO (light green; cubic PDF 00-047-1049 and rhombohedral PDF 04-011-2340), Pd (dark gold; cubic PDF 00-005-0681), and PdO (light gold; tetragonal PDF 00-041-1107). Cubic and rhombohedral phases show only slight changes in relative intensity ratios.

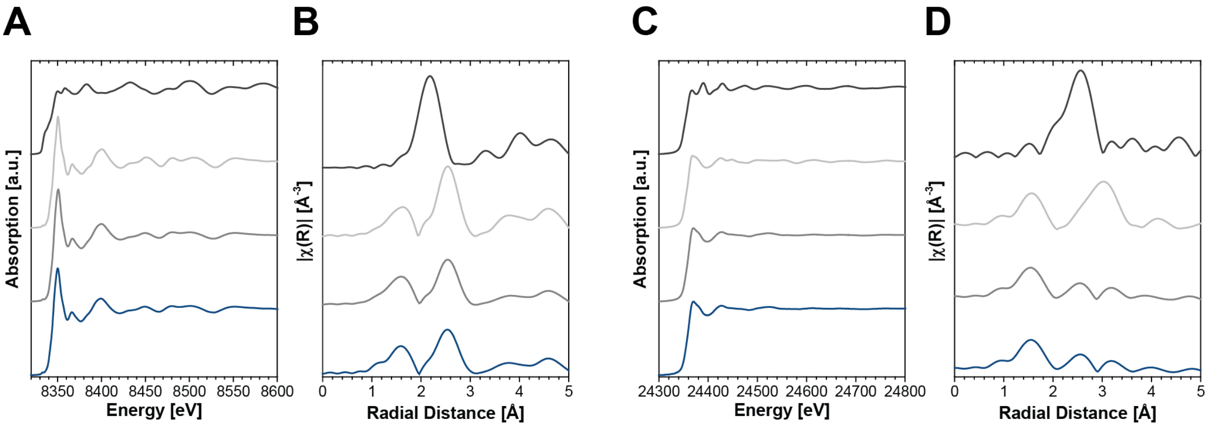

Figure S2: *Ex situ* X-ray absorption spectroscopy (XAS) spectra and corresponding Fourier transforms at the (**A**,**B**) Ni K-edge: nanometer-sized supported catalysts ($Pd_{0.06}Ni_{0.94}O/SiO_2$ and NiO/SiO_2) and bulk reference materials (NiO and metallic Ni). (**C**,**D**) Pd K-edge: nanometer-sized supported catalysts ($Pd_{0.06}Ni_{0.94}O/SiO_2$ and PdO/SiO_2) and bulk reference materials (PdO and metallic Pd). Spectra were normalized by the edge jump and corrected for edge position. The Fourier transforms were obtained using the following parameters: Ni-edge (k:3-12Å; R_{bkg}:1.0 Å) and Pd-edge (k:4.5-11Å; R_{bkg}:1.0 Å).

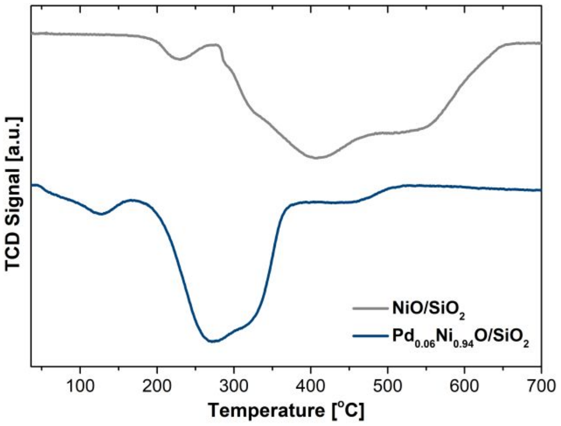

Figure S3: Temperature-programmed reduction (TPR) profile in 5 vol.% H_2/Ar of $Pd_{0.06}Ni_{0.94}O/SiO_2$ and NiO/SiO_2. Heated with 5K/min temperature ramps.

Table S3: Catalytic performance of the catalysts tested under CO_2 hydrogenation conditions (CO_2/H_2 ratio: 1:4; gas-hourly space velocity (GHSV): $1.5.10^5$ h^{-1}; catalyst mass of 20 mg; pressure of 1 atm)

$Pd_{0.06}Ni_{0.94}O/SiO_2$	*Conversion of CO_2 [mol.%]*	*Metal-based Activity [$mol_{Product}/(s.mol_{Metal}$]*	*Selectivity towards CH_4 [mol.%]*	*Selectivity towards CO [mol.%]*
250°C	0.5	$6.2 \cdot 10^{-4}$	71	29
400°C	23.7	$2.9 \cdot 10^{-2}$	74	26

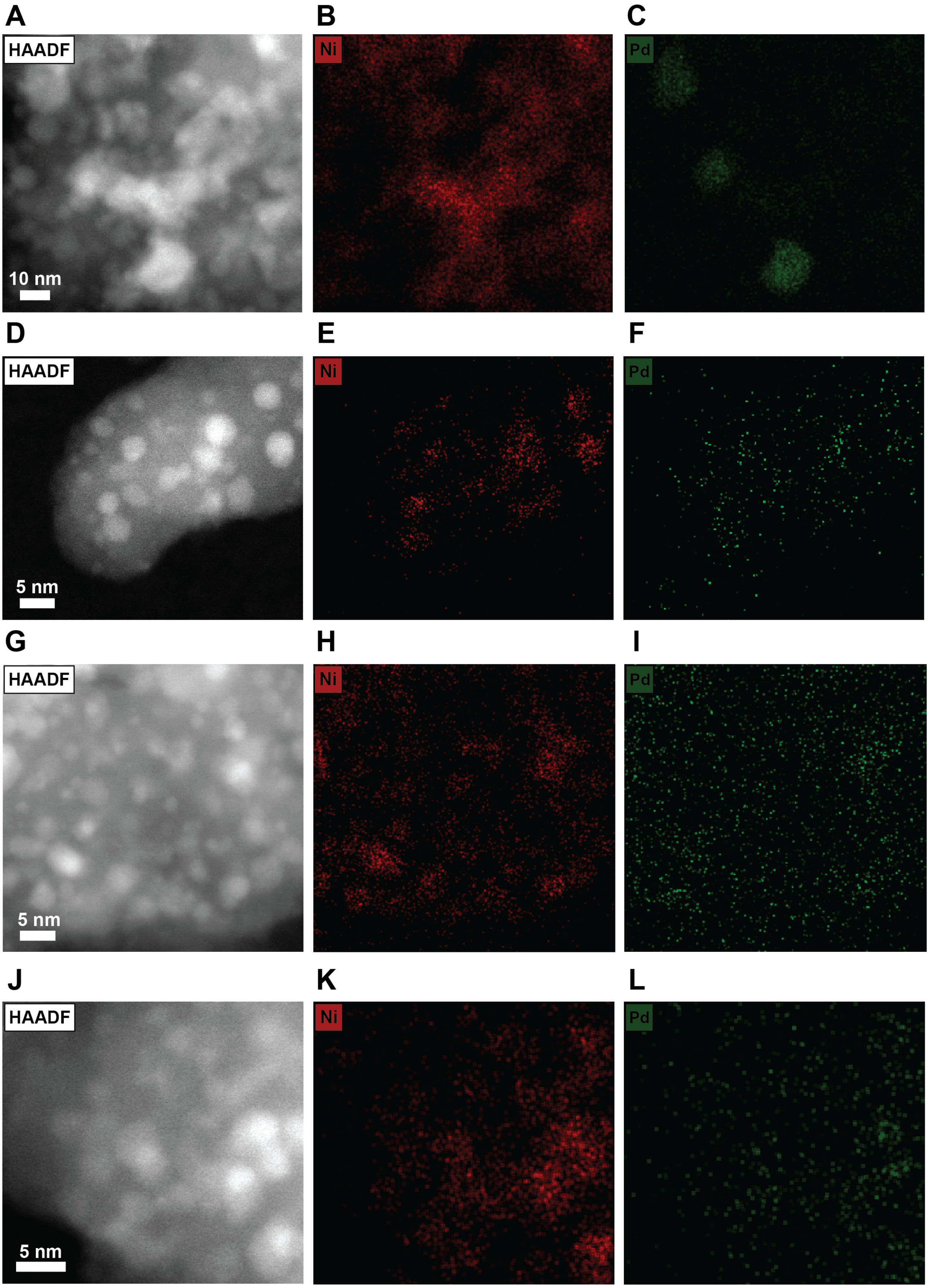

Figure S4: Scanning transmission electron microscopy coupled with energy-dispersive X-ray spectroscopy (STEM-EDX) imaging of $Pd_{0.06}Ni_{0.94}O/SiO_2$. Analysis areas containing Pd-rich nanoparticles (**A**-**C**) and Ni-rich nanoparticles (**D**-**F**, **G**-**I,** and **J**-**L**). Only a few areas of the catalyst contain Pd-rich nanoparticles, while most areas comprise of Ni-rich nanoparticles.

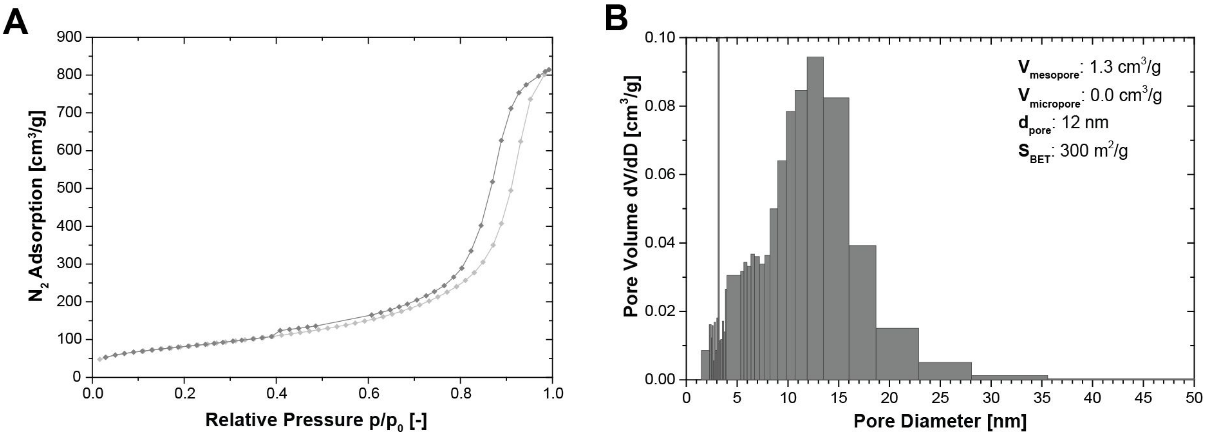

Figure S5: (**A**) Adsorption and desorption isotherms obtained from N_2 physisorption. (**B**) Pore size distribution of the $Pd_{0.06}Ni_{0.94}O/SiO_2$ obtained from by the Barrett-Joyner-Halenda (BJH) method.[17] Values extracted from the desorption isotherm: $V_{mesopore}$: mesopore volume, $V_{micropore}$: micropore volume, d_{pore}: average pore diameter, and S_{BET}: Brunauer–Emmett–Teller surface area.

Atom Probe Specimen Preparation

Silica-embedding of nanoparticles was performed by wetness impregnation. The catalyst was pre-dried under stirring at 230 °C for 2 h under dynamic vacuum (2-3 mbar). Subsequently, the powder was cooled to room temperature and impregnated drop-by-drop with an excess of sodium silicate solution (Sigma Aldrich, 40 wt.% in water). The volume added was based on ten-fold the pore volume of the catalyst. Drying was performed at room temperature at ambient pressure. Dried samples were calcined in tubular ovens in N_2 with a heating rate (r_{Heat}) of 2 °C/min to a maximum temperature (T_{max}) of 400°C, and a duration at maximum temperature (t_{max}) of 2 h. Lift-out FIB milling procedures were performed as described in the Materials section.

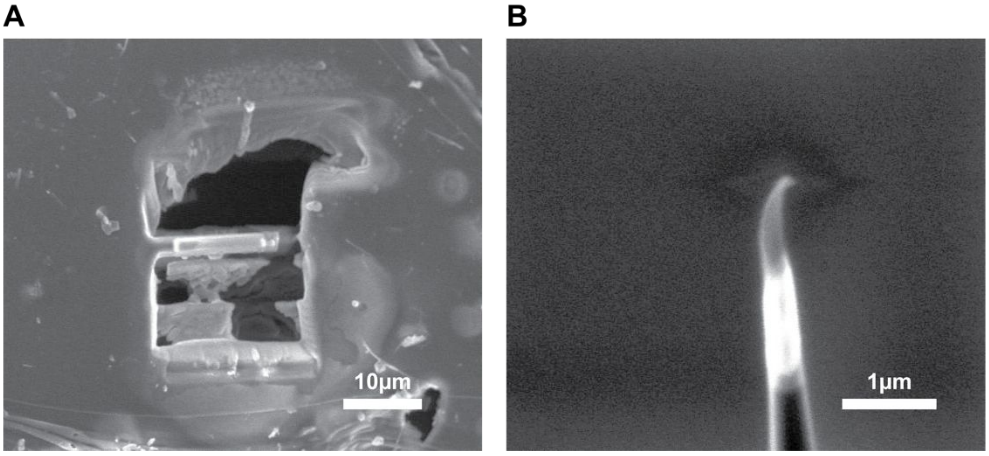

Figure S6: Focused ion beam (FIB) milling issues for silica-embedded nanoparticles. (**A**) Trench cut for lift-out procedure. (**B**) Final tip shape.

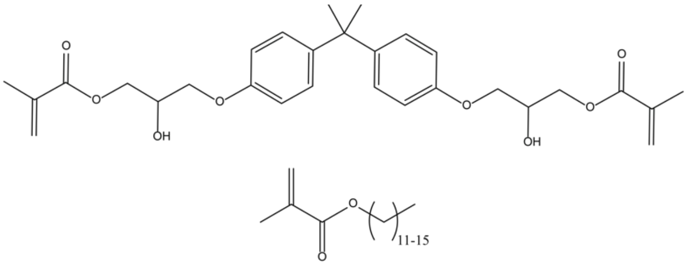

Figure S7: Main components of the LR White Resin (acrylic resin) (**B**).

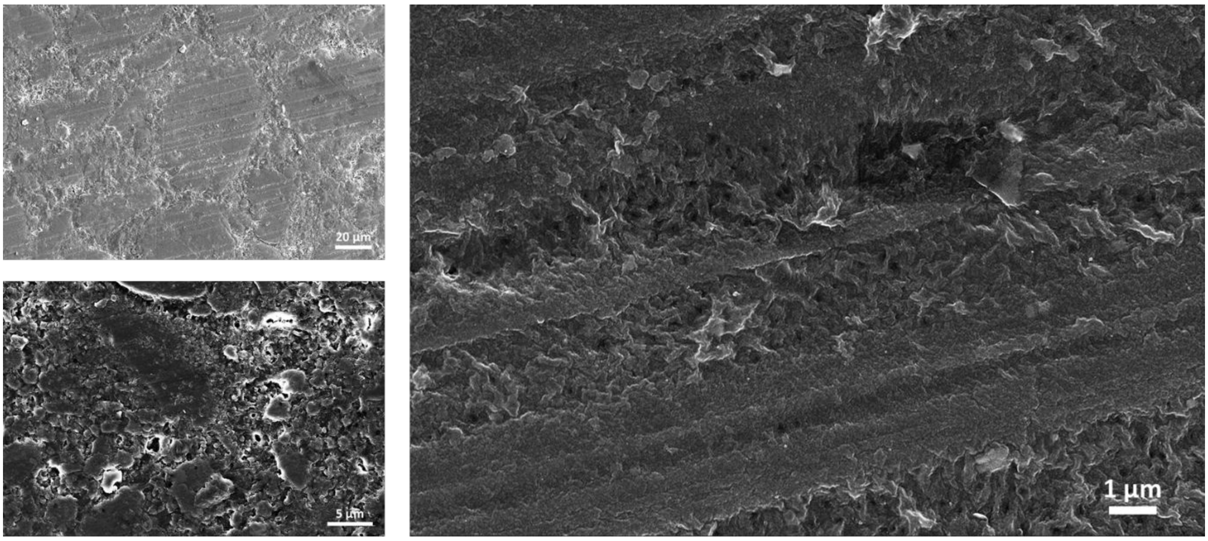

Figure S8: Scanning electron microscopy (SEM) images of the cross section obtained after diamond cutting.

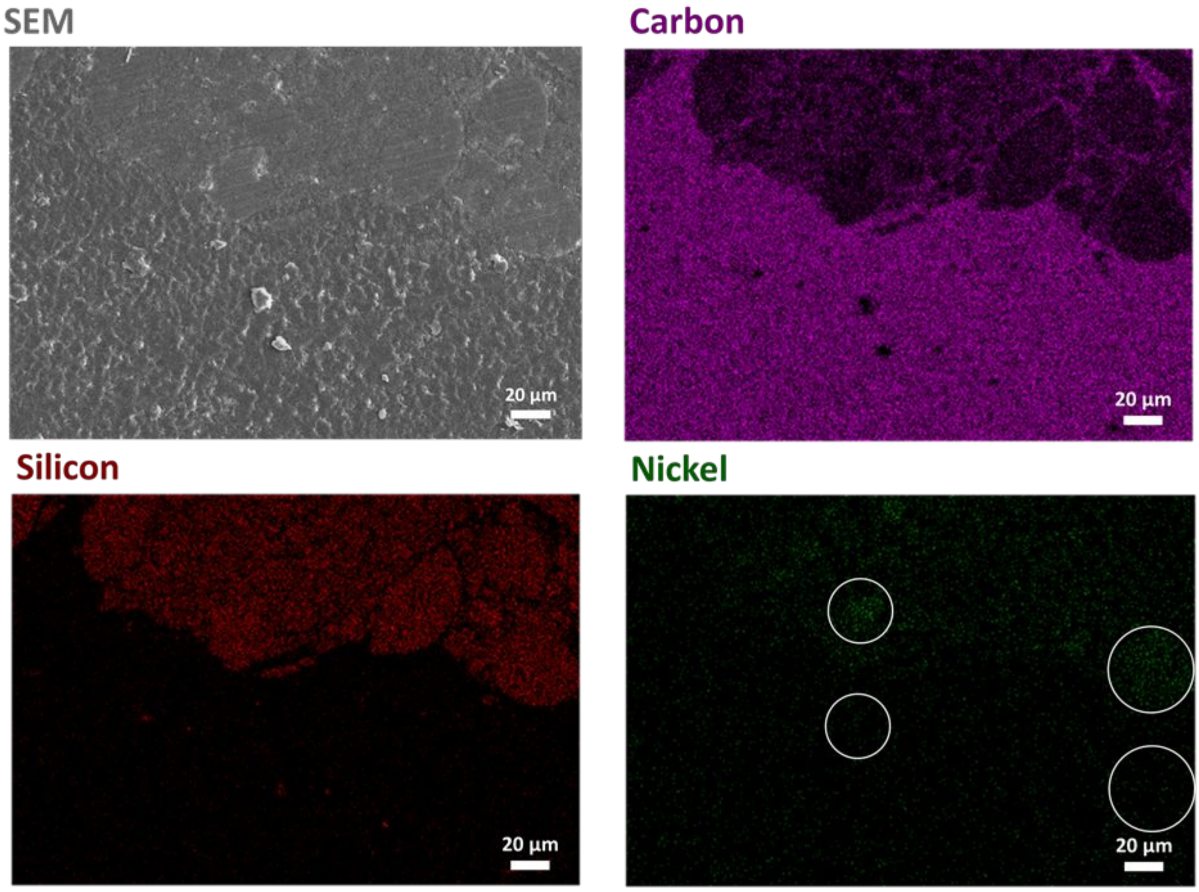

Figure S9: Scanning electron microscopy energy-dispersive X-ray spectroscopy (SEM-EDX) images of the cross section for identifying the lift-out position. Circles emphasize the differences in EDX signal between nickel-rich areas (top two circles) and resin matrix (bottom two circles).

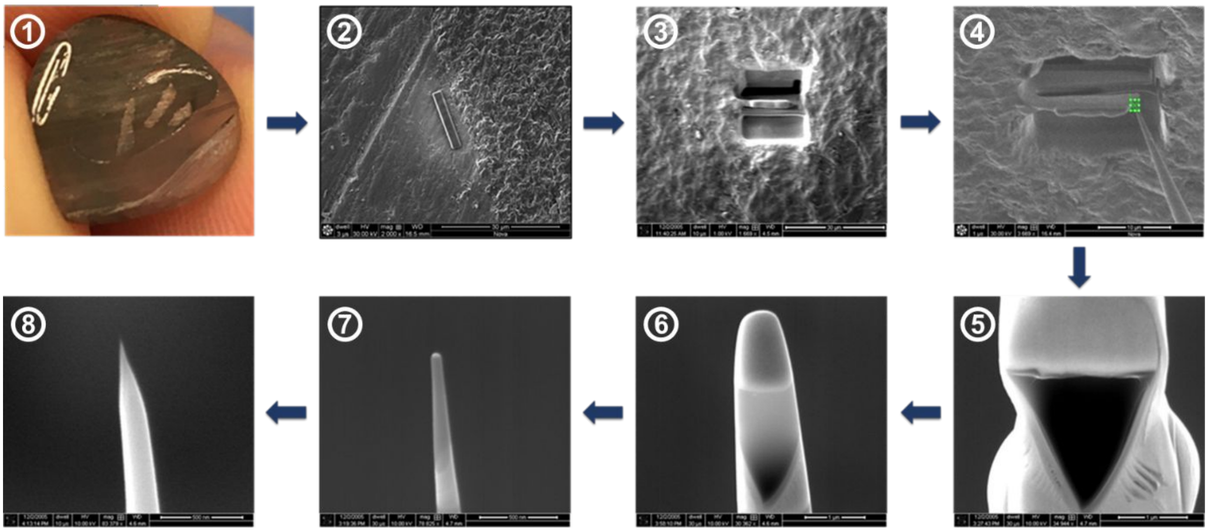

Figure S10: Atom probe specimen preparation: (1) cross-section of the resin-impregnated stub containing the three impregnated compacted catalyst slivers, (2) lift-out position protected by vapor deposited platinum strip, (3) trench cut gallium ion milling to cut out the wedge-shaped sample, (4) detachment of the wedge and transfer onto silicon micro-tip coupon, (5) platinum welded wedge-shaped sample on top of micro-tip coupon, (6) first annular focused ion beam (FIB) milling step with 30keV, (7) second annular FIB milling step with 2 keV and (8) final atom probe specimen.

Selected area electron diffraction (SAED) analysis (**Figure S11**) suggests the presence of a cubic crystal phase, as can be seen for the sequence of diffraction lines expected for the space group Fm-3m. Measured lattice spacings and assigned lattice planes were 2.53 (111), 2.18 (200), 1.55 (220), and 1.31 Å (311). While three different areas of the catalyst were analyzed, only slight differences of < 0.03 Å were found. Although slightly deviating from the expected NiO lattice spacing for the 111 planes of 2.41 Å (PDF 00-047-1049), metallic Ni would give a value of 2.03 Å (PDF 00-004-0850). This further corroborates the presence of oxide nickel nanoparticles, as was found for XRD, XAS, and TPR.

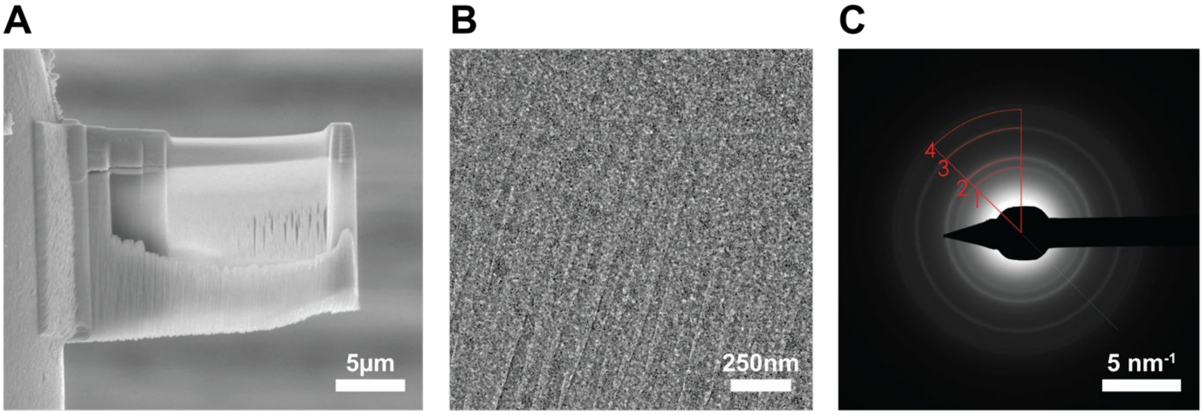

Figure S11: (**A**) Scanning electron microscopy (SEM) image of the focus ion beam milled lamella prepared from the resin-impregnated stub of $Pd_{0.06}Ni_{0.94}O/SiO_2$. (**B**) Scanning transmission electron microscopy (STEM) image of the analysis area within the lamella for selected area electron diffraction (SAED). (**C**) SAED diffraction pattern of the analysis area.

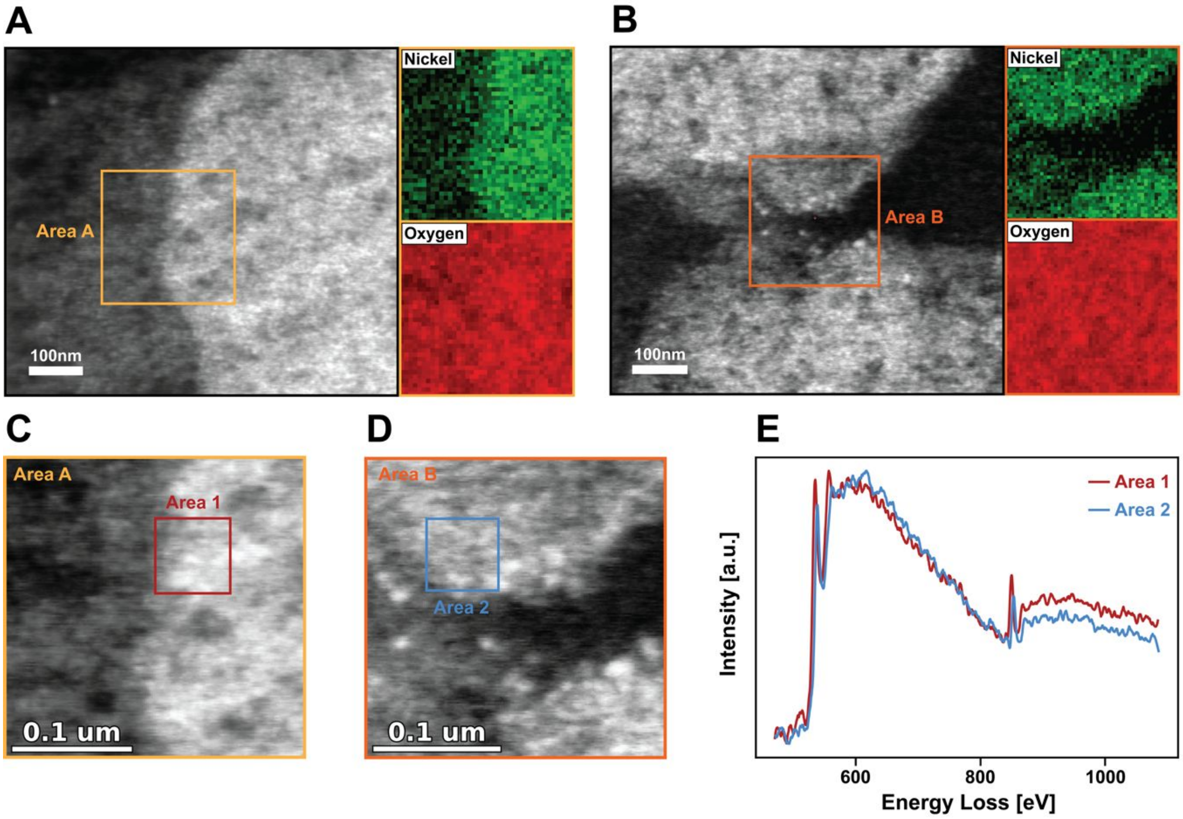

Figure S12: Scanning transmission electron microscopy coupled with electron energy loss spectroscopy (STEM-EELS) of the focus ion beam milled lamella prepared from the resin-impregnated stub of $Pd_{0.06}Ni_{0.94}O/SiO_2$. (**A** and **B**) Elemental maps of nickel (green, top right panels) and oxygen (red, bottom right panels) for two representative areas of the resin-embedded catalyst materials. (**E**) Core-loss spectra of the displayed analysis areas (**C** and **D**).

Atom Probe Tomography

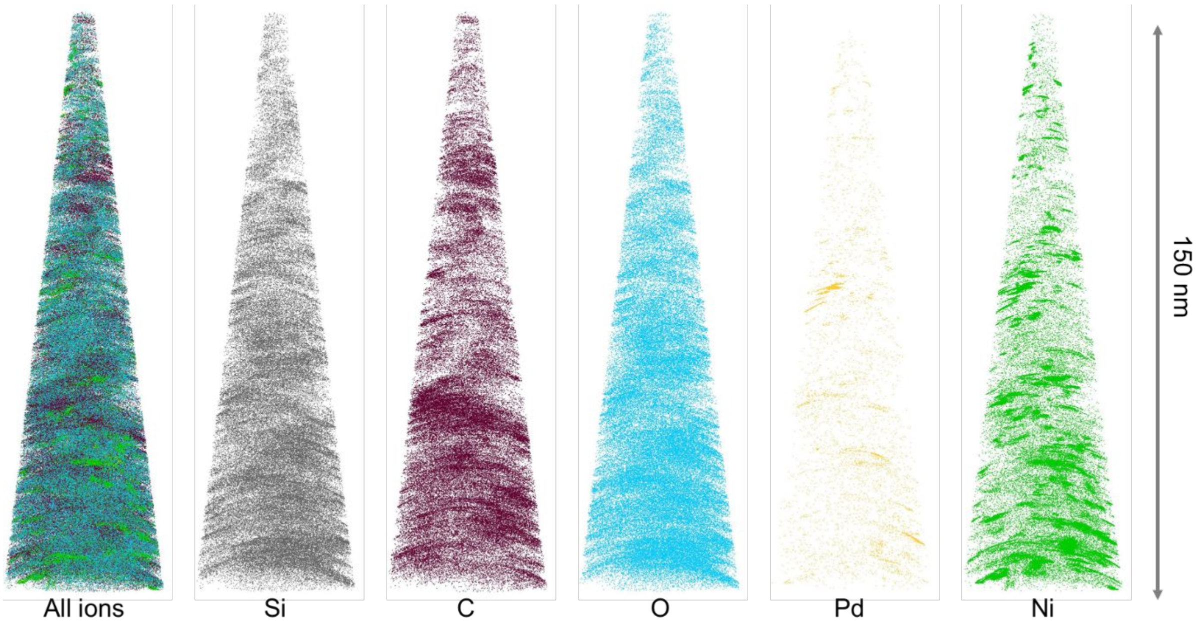

Figure S13: Three-dimensional reconstruction for different elements. Sum of mass fragments corresponding to certain elements (**Movie S1**).

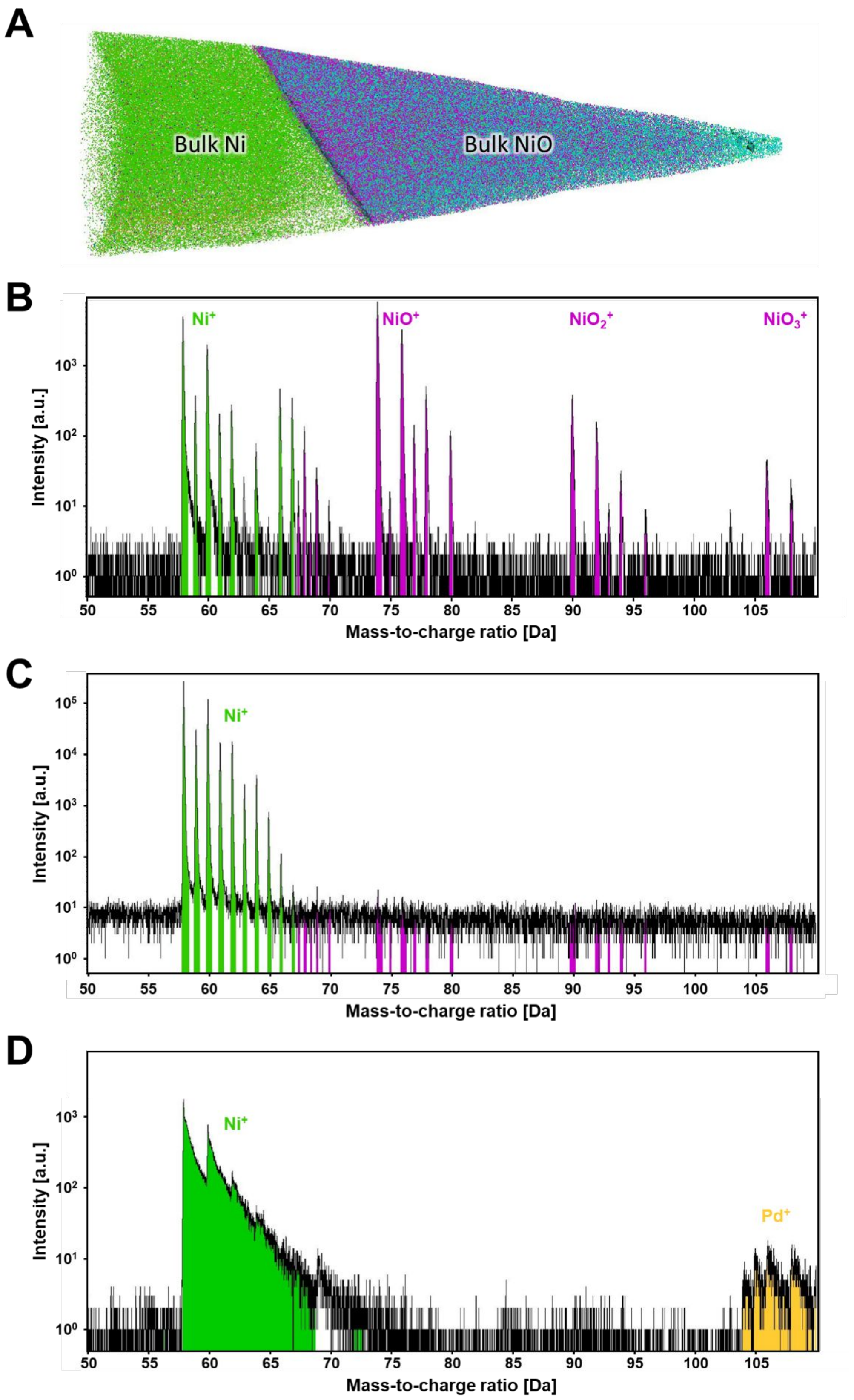

Figure S14: (**A**) Reconstruction of reference material containing bulk metallic nickel and nickel oxide.[2] Mass spectrometry (MS) pattern with corresponding field-evaporated ionic species: (**B**) bulk nickel oxide (**C**) bulk metallic nickel and (**D**) resin-impregnated $Pd_{0.06}Ni_{0.94}O/SiO_2$ after clustering. Parameters for cluster analysis: solute atoms: Pd and Ni, d_{max}:0.3nm, N_{min}: 16, L,E: 0.8 * d_{max}.

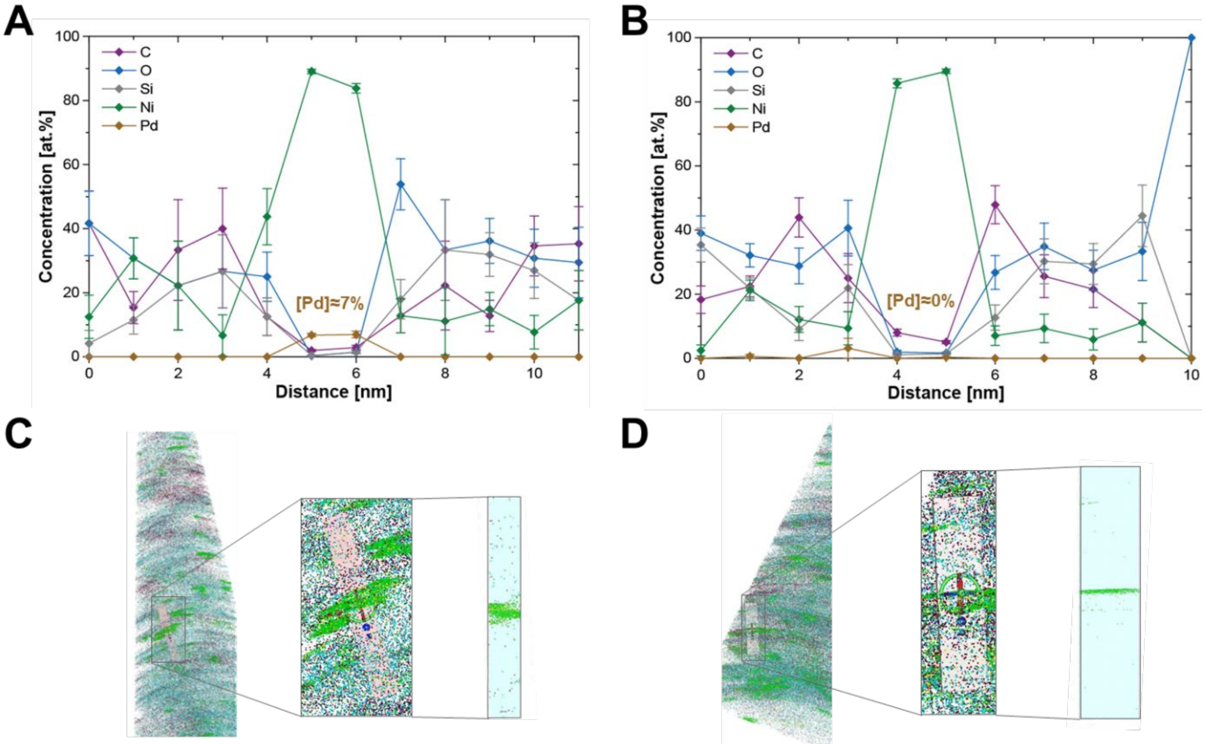

Figure S15: One-dimensional concentration profiles (1DCP) for two high-concentration areas of the reconstructed dataset $Pd_{0.06}Ni_{0.94}O/SiO_2$. (**A**,**B**) 1DCP for the sum of mass fragments corresponding to the element as a function of distance performed as depicted in (**C**,**D**).

For a more detailed description of cluster analysis in general, the algorithm applied as well as its involved variables we refer to the literature.[18]

While cluster analysis is a highly valuable tool to assess APT datasets, its results are highly influenced by the variables used. Therefore, care has to be taken for choosing appropriate variables. While cluster size distribution and cluster count distribution are helpful metrics for variable choice, these were not applicable to the data set. Hence, we have opted for sensitivity analysis guided by visual inspection of the data set according to electron microscopy. For the sensitivity analysis, mostly the effect of d_{max} was investigated. As this variable describes the maximum distance between atoms within clusters, setting this value too low will separate clusters while too high values merge clusters. Furthermore, the result of clustering (**Figure S16** and **Movie S2**), showcases that a tradeoff has to be made between obtained spatial elemental information and cluster separation. For d_{max}=0.1 nm, clusters are separated into sub-clusters while only < 20at.% of the solute atoms (metals) within the dataset are described by cluster analysis (**Figure S17**). In contrast for d_{max}=0.5 nm, clusters are merged, and although around 70at.% of solute atoms are described by cluster analysis, also around 10at.% of matrix atoms are incorporated into the clusters.

When looking at the range of parameters varied, a full image of cluster analysis can be gathered. As a function of d_{max}, the obtained number of clusters, cluster size based on solute atoms and cluster volume, as well as most importantly, histograms displaying the heterogeneity in size and composition are compiled in **Figure S18**. Notably, the change in N_{min} illustrates, that mostly the fraction of small clusters is increased while the overall trends hold (**Figure S18D** and **S18E**). For d_{max}, a somewhat insensitive plateau of values is found around 0.2-0.4 nm. Further looking into the clustering performance, significant cluster separation issues were monitored for 0.4 nm. Although these issues remain even for the bottom part of the reconstruction of 0.2 nm, additionally separation into sub-clusters was observed for the top part of the reconstruction. As a compromise between those issues, a d_{max} value of 0.3 nm was chosen although clustering remains imperfect. Aiming to maximize spatial compositional information, this parameter seems a sensible choice.

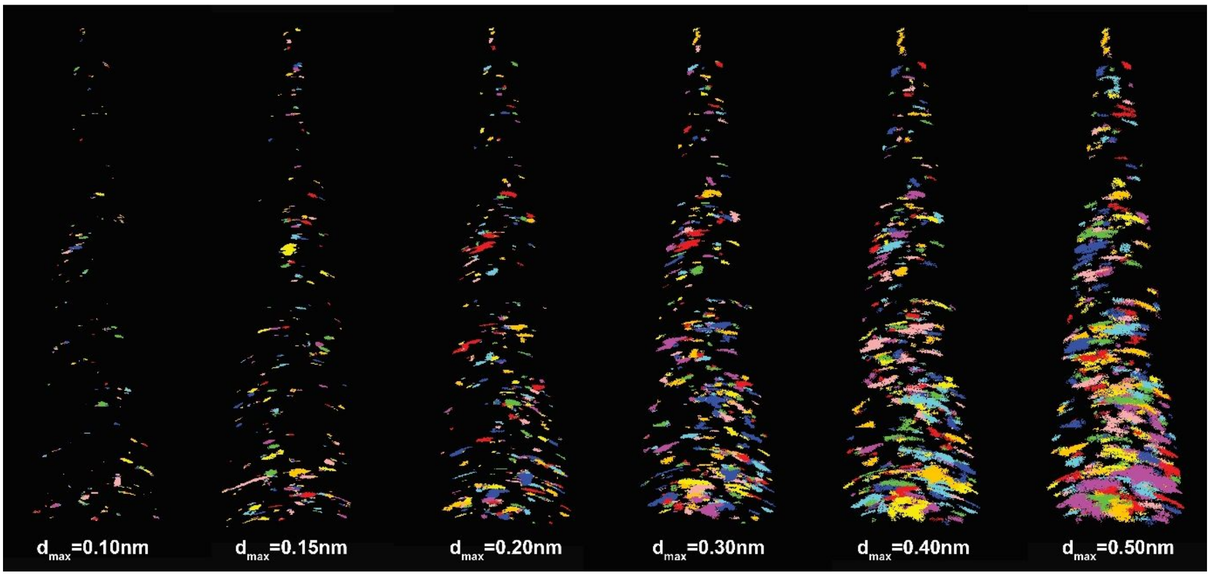

Figure S16: Clustering results obtained for different d_{max} values. Repetitive use of a limited number of different colors might compromise the reader's ability to distinguish clusters in the 2D projection. Therefore, for more detailed inspection of the clustering, the reader is referred to the supporting video section (**Movie S2**).

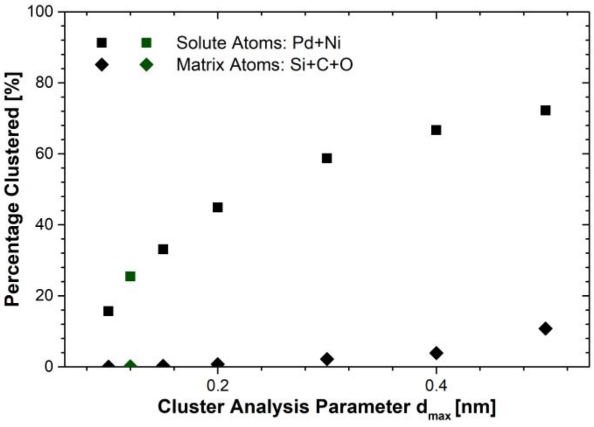

Figure S17: Percentage of mass fragments of solute atoms (Pd and Ni) and matrix atoms (Si, C, and O) captured by cluster analysis. Parameters: solute atoms: Pd and Ni, d_{max}: 0.1-0.5 nm, N_{min}: 16 (10, green), L,E: 0.8 * d_{max}.

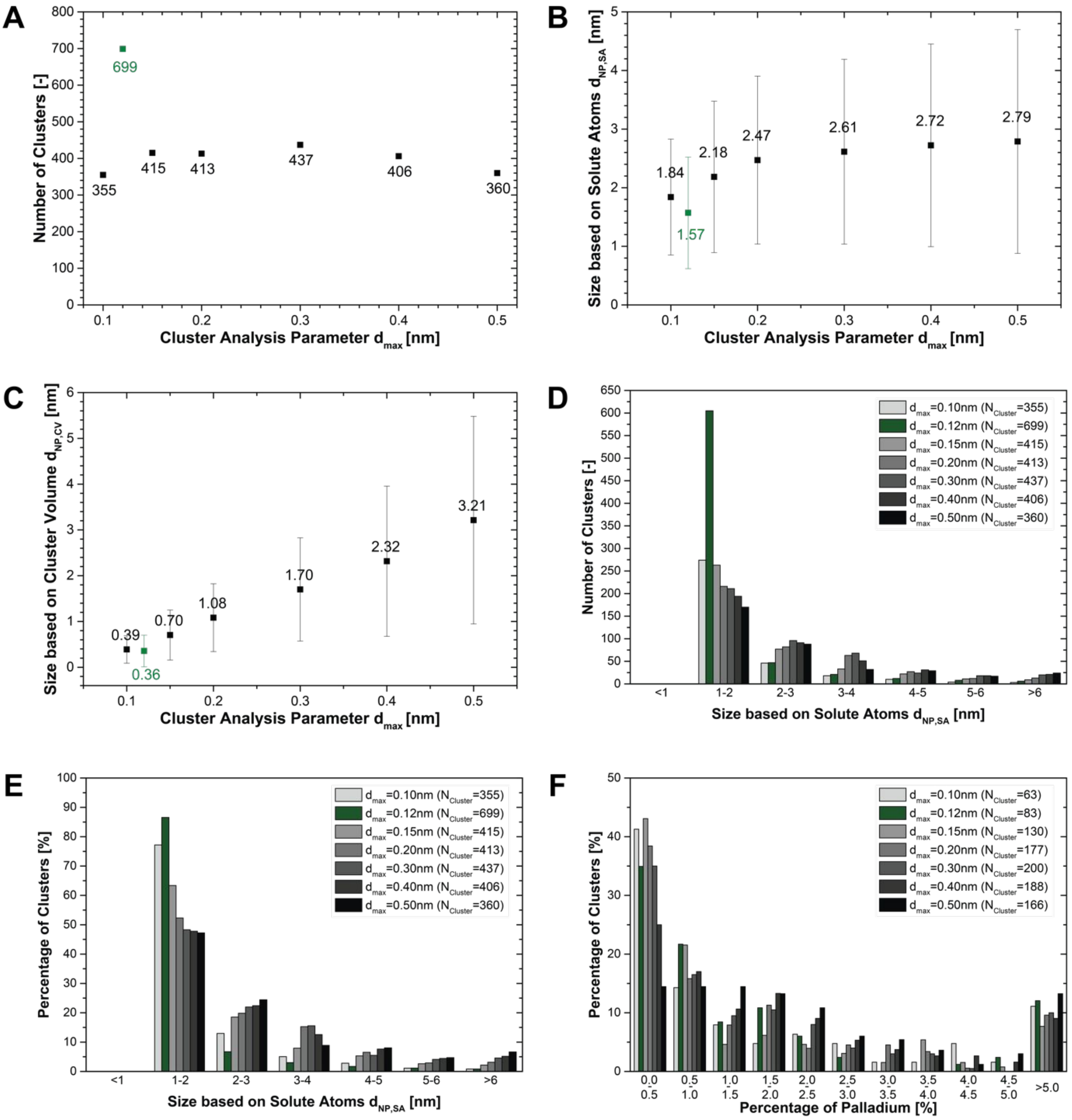

Figure S18: Sensitivity analysis for the cluster analysis parameters d_{max} and N_{min} using the maximum separation method. Parameters: solute atoms: Pd and Ni, d_{max}: 0.1-0.5 nm, N_{min}: 16 (10, green), L,E: 0.8 * d_{max}. (**A**) Number of obtained clusters. (**B**,**C**) Average cluster sizes calculated by spherical nanoparticle model based on number of solute atoms (**B**, see Equation **(1)**) or cluster volume (**C**, see Equation **(2)**). (**D**,**E**) Particle size histograms obtained from solute atoms as total number of clusters (**D**) or percentage of clusters (**E**). (**F**) Composition histograms obtained from solute atoms assuming the nanoparticles only contain Ni and Pd.

$$d_{NP,SA} \approx \sqrt[3]{\frac{\frac{6*1}{\varepsilon_{detector}} * N_{total} * \left(\frac{N_{Ni}}{N_{total}} * M_{NiO} + \frac{N_{Pd}}{N_{total}} * M_{PdO}\right)}{\pi * N_A * \left(\frac{N_{Ni}}{N_{total}} * \rho_{NiO} + \frac{N_{Pd}}{N_{total}} * \rho_{PdO}\right)}} \quad \textbf{(1)}$$

$$d_{NP,CV} \approx \sqrt[3]{\frac{6 * V_{cluster}}{\pi}} \quad \textbf{(2)}$$

Symbols: $d_{NP,SA}/d_{NP,CV}$: cluster size calculated by spherical nanoparticle model based on solute atoms/cluster volume, $\varepsilon_{detector}$: detector efficiency, $N_{total}/N_{Ni}/N_{Pd}$: number of total (solute)/nickel/palladium atoms per cluster, M_{NiO}/M_{PdO}: molar mass of nickel oxide/palladium oxide, ρ_{NiO}/ρ_{PdO}: bulk density of nickel oxide/palladium oxide, N_A: Avogadro constant, $V_{cluster}$: cluster volume

For compositional analysis, the ranging of mass fragments was optimized via clustering (**Figure S19**). Thereby, spectral interferences between mass fragments (e.g., $^{60}Ni^+$ and $^{28}Si^{16}O_2^+$) were limited by excluding matrix elements. Although thermal tails persist after clustering, originating from the non-conductive nature of the specimen,[18-20] overlaps between thermal tails of different mass fragments were limited. However, although quantification of matrix elements such as Si, C, and O is difficult, quantification of metal ratios is generally feasible within alloys.[21] Due to the isolation of metal-related mass fragments by clustering, quantification is mostly affected by evaporation field differences. However, in the case of Pd and Ni only slight differences are expected enabling sufficiently accurate quantification (Pd^+: 37 V/nm, Ni^+: 35 V/nm).

Furthermore, mislabeling was addressed as Si-based mass fragments might be interpreted as Ni. Therefore intensity ratios for the MS peaks ascribed to Ni were compared with the nickel isotope ratio. Given that the peak with m/z=58 is not interfered via Si-based mass fragments, we use the following peak as reference to assess the spectral inferences between $^{28}Si^{16}O_2^+$ and $^{60}Ni^+$. Peak ratios were obtained via i) the integrals of the peaks, ii) the intensity maxima, and iii) the averaged values around the maxima. Ratios of 2.64±0.17 were obtained for the clustered data (**Figure S19B**), in line with the isotope ratio of 2.60, confirming limited mislabeling for the clustering result.

To assess the losses related to the mass ranging of Ni-based on Ni^+ (performed for all data analysis), Ni^{2+} species were quantified, as those represent the second most abundant ionic Ni species, given that intensities for Ni^{3+} and NiO^{n+} species were low. When calculating the ratio between m/z=58 and m/z=29, a value of around 14 was obtained for the reconstructed data. Although the peak at m/z=29 comprises of $^{58}Ni^{2+}$, $^{29}Si^+$, $^{28}Si^1H^+$, $^{12}C^{16}O^1H^+$, the sum of these species only makes up 8% of the intensity of $^{58}Ni^+$. Therefore, by neglecting divalent nickel species less than 7% of nickel species were lost.

Nevertheless, when larger distances between solute atoms d_{max}=0.5nm were chosen, intensity ratios of 2.36±0.11 were obtained, evidencing spectral interferences, as present also for the reconstructed data with a ratio of 1.8±0.3 (representing around 10% of the Ni-labeled species). In combination with the comparable size distribution obtained from cluster analysis (**Figure 3C**), we believe that the choice of cluster parameters enables the representative compositional analysis of the clusters.

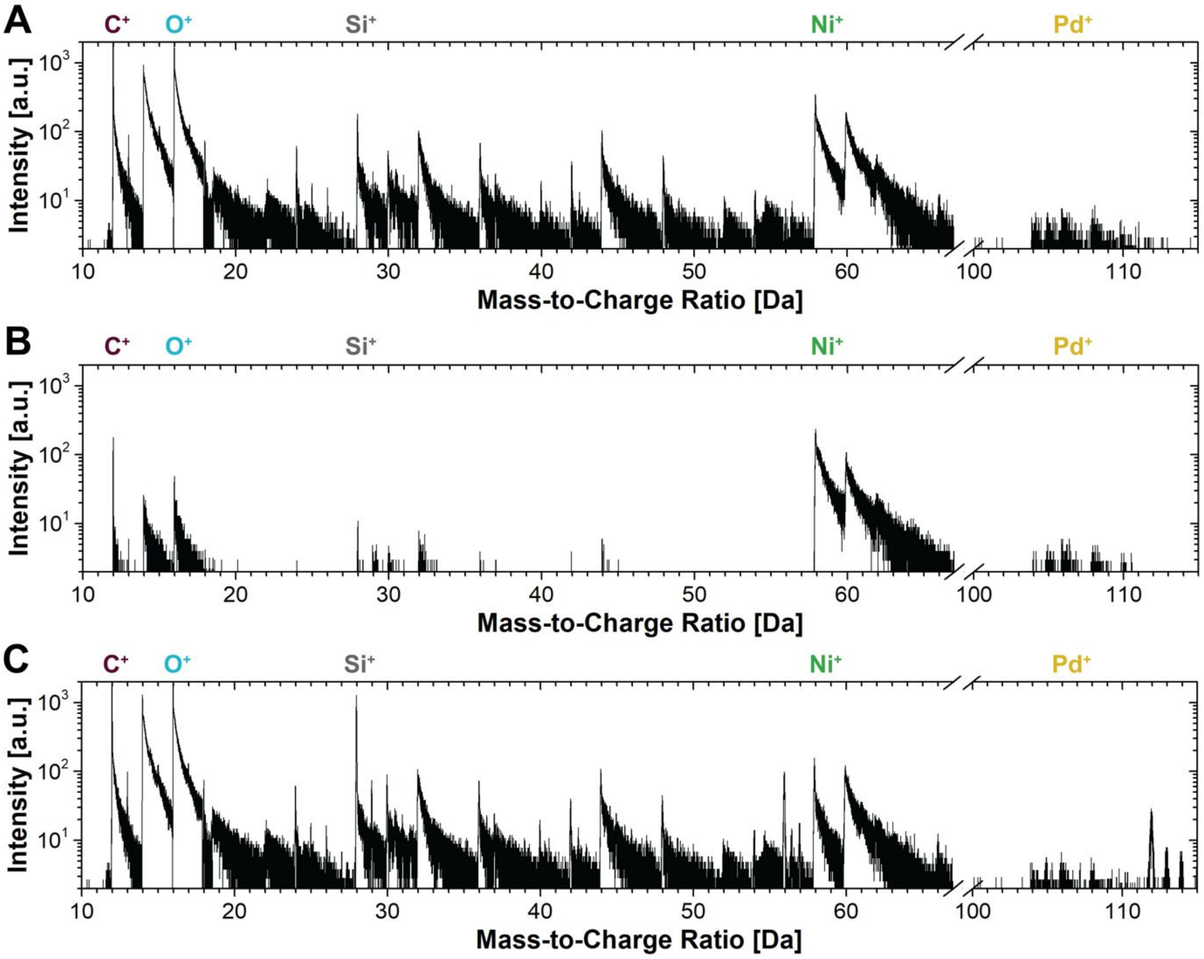

Figure S19: Mass spectrometry (MS) pattern for the reconstructed data set (**A**) and after cluster analysis of the identified clusters (**B**) and the matrix (**C**). Parameters for cluster analysis: solute atoms: Pd and Ni, d_{max}:0.3nm, N_{min}: 16, L,E: 0.8 * d_{max}. Assignment of the single-charged main fragments within the sample.

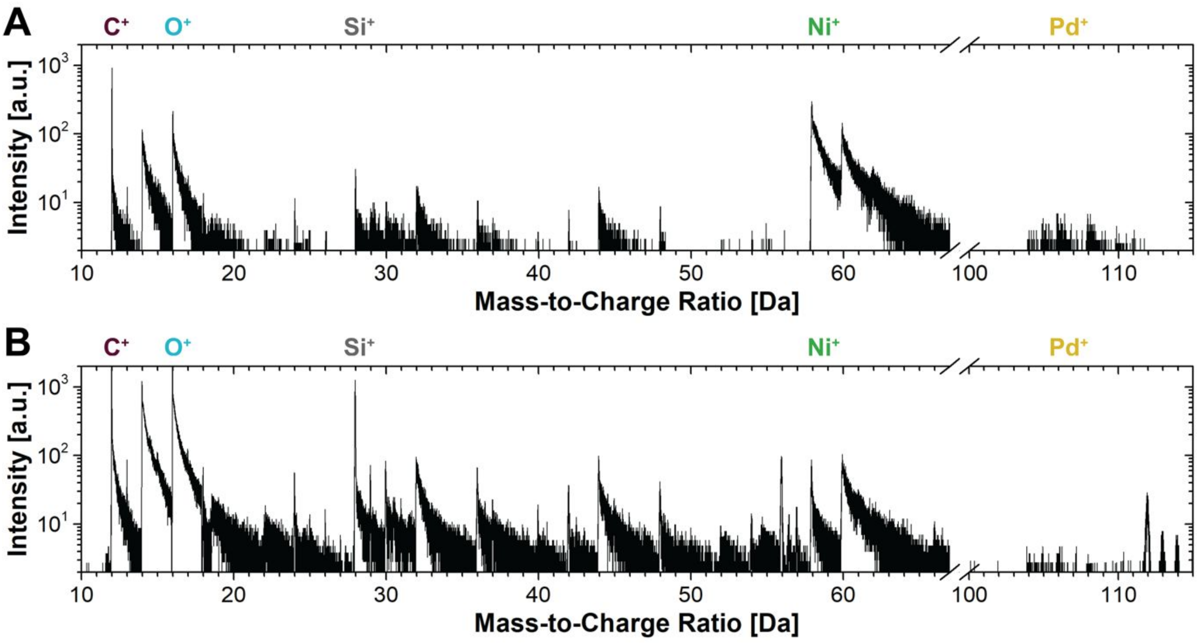

Figure S20: Mass spectrometry (MS) pattern after cluster analysis of the identified clusters (**A**) and the matrix (**B**) obtained from non-accurate relaxed parameter choice. Parameters for cluster analysis: solute atoms: Pd and Ni, d_{max}:0.5nm, N_{min}: 16, L,E: 0.8 * d_{max}. Assignment of the single-charged main fragments within the sample

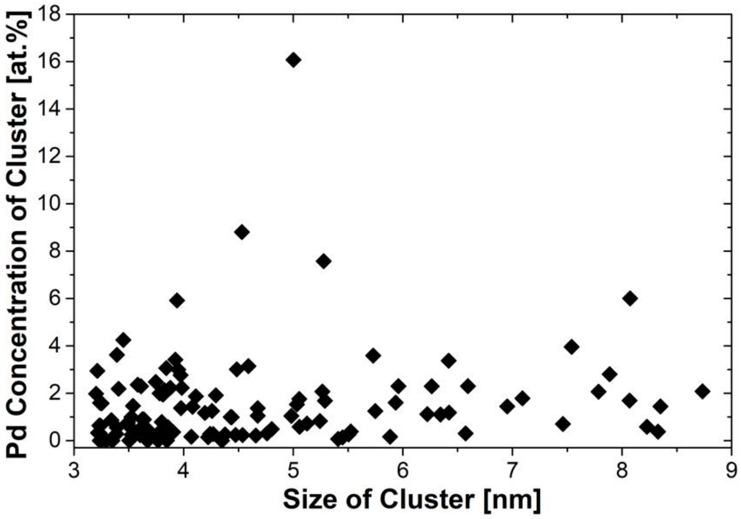

Figure S21: Correlation between cluster size and Pd concentration ($N_{Pd,cluster}/(N_{Ni,cluster}+N_{Pd,cluster})$) for clusters with $N_{Ni,cluster}+N_{Pd,cluster}$ > 300. Parameters for cluster analysis: solute atoms: Pd and Ni, d_{max}:0.3nm, N_{min}: 16, L,E: 0.8 * d_{max}.

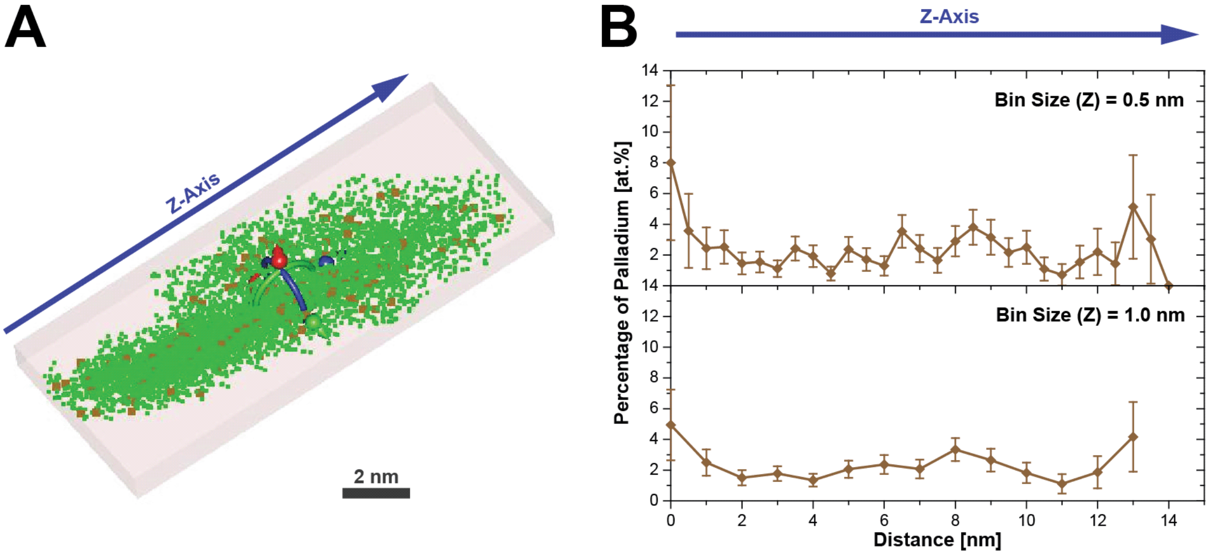

Figure S22: One-dimensional concentration profiles (1DCP) on the identified clusters from cluster analysis. Illustrated for the cluster of intermediate palladium concentration (18507 atoms, 2.1 at.% Pd) as depicted in **Figure 4D** (second from the bottom). (**A**) Probed volume encloses the whole cluster. (**B**) 1DCP in z direction with a bin width of 0.5 nm (top) and 1.0 nm (bottom). Error bars calculated from error propagation (see Equation **(3)**).

$$\sigma_{[Pd]} \approx \sqrt{\frac{{\sigma_{Pd}}^2 * {N_{Ni}}^2 + {\sigma_{Ni}}^2 * {N_{Pd}}^2}{(N_{Pd} + N_{Ni})^4}} \quad \text{for} \quad [Pd] = \frac{N_{Pd}}{N_{Pd} + N_{Ni}} \tag{3}$$

Symbols: N_{Ni}/N_{Pd}: number of nickel/palladium atoms within fragment of the cluster (bin width), σ_{Pd}/σ_{Ni}: corresponding standard deviation, $[\mathrm{Pd}]$: concentration of palladium within fragment of the cluster, $\sigma_{[Pd]}$: standard deviation of the palladium concentration based on error propagation

Bibliography

(1) Thompson, K.; Lawrence, D.; Larson, D.; Olson, J.; Kelly, T.; Gorman, B., In situ site-specific specimen preparation for atom probe tomography. *Ultramicroscopy* **2007,** *107* (2-3), 131-139.

(2) Poplawsky, J. D.; Pillai, R.; Ren, Q.-Q.; Breen, A. J.; Gault, B.; Brady, M. P., Measuring oxygen solubility in Ni grains and boundaries after oxidation using atom probe tomography. *Scripta Materialia* **2022,** *210*, 114411.

(3) Yang, Q.; Danaie, M.; Young, N.; Broadley, V.; Joyce, D.; Martin, T.; Marceau, E.; Moody, M.; Bagot, P. A., Atom probe tomography of Au–Cu bimetallic nanoparticles synthesized by inert gas condensation. *The Journal of Physical Chemistry C* **2019,** *123* (43), 26481-26489.

(4) Felfer, P.; Li, T.; Eder, K.; Galinski, H.; Magyar, A.; Bell, D.; Smith, G.; Kruse, N.; Ringer, S.; Cairney, J., New approaches to nanoparticle sample fabrication for atom probe tomography. *Ultramicroscopy* **2015,** *159*, 413-419.

(5) Felfer, P.; Benndorf, P.; Masters, A.; Maschmeyer, T.; Cairney, J. M., Revealing the distribution of the atoms within individual bimetallic catalyst nanoparticles. *Angewandte Chemie International Edition* **2014,** *53* (42), 11190-11193.

(6) Xiang, W.; Yang, N.; Li, X.; Linnemann, J.; Hagemann, U.; Ruediger, O.; Heidelmann, M.; Falk, T.; Aramini, M.; DeBeer, S., 3D atomic-scale imaging of mixed Co-Fe spinel oxide nanoparticles during oxygen evolution reaction. *Nature Communications* **2022,** *13* (1), 179.

(7) Yang, Q.; Joyce, D.; Saranu, S.; Hughes, G.; Varambhia, A.; Moody, M.; Bagot, P., A combined approach for deposition and characterization of atomically engineered catalyst nanoparticles. *Catalysis, Structure & Reactivity* **2015,** *1* (3), 125-131.

(8) Larson, D. J.; Giddings, A.; Wu, Y.; Verheijen, M.; Prosa, T.; Roozeboom, F.; Rice, K.; Kessels, W.; Geiser, B.; Kelly, T., Encapsulation method for atom probe tomography analysis of nanoparticles. *Ultramicroscopy* **2015,** *159*, 420-426.
(9) Barroo, C.; Akey, A. J.; Bell, D. C., Aggregated nanoparticles: Sample preparation and analysis by atom probe tomography. *Ultramicroscopy* **2020,** *218*, 113082.
(10) Li, T.; Bagot, P. A.; Christian, E.; Theobald, B. R.; Sharman, J. D.; Ozkaya, D.; Moody, M. P.; Tsang, S. E.; Smith, G. D., Atomic imaging of carbon-supported Pt, Pt/Co, and Ir@ Pt nanocatalysts by atom-probe tomography. *ACS Catalysis* **2014,** *4* (2), 695-702.
(11) Vegard, L.; Dale, H., An investigation of mixed crystals and alloys. *Zeitschrift für Kristallografie* **1928,** *67*, 148-161.
(12) Nash, A.; Nash, P., The Ni−Pd (Nickel-Palladium) system. *Bulletin of Alloy Phase Diagrams* **1984,** *5* (5), 446-450.
(13) Chen, Y.-g.; Tomishige, K.; Yokoyama, K.; Fujimoto, K., Promoting effect of Pt, Pd and Rh noble metals to the $Ni_{0.03}Mg_{0.97}O$ solid solution catalysts for the reforming of CH_4 with CO_2. *Applied Catalysis A: General* **1997,** *165* (1-2), 335-347.
(14) Tomishige, K.; Kanazawa, S.; Sato, M.; Ikushima, K.; Kunimori, K., Catalyst design of Pt-modified Ni/Al_2O_3 catalyst with flat temperature profile in methane reforming with CO_2 and O_2. *Catalysis Letters* **2002,** *84* (1), 69-74.
(15) Wong, A.; Liu, Q.; Griffin, S.; Nicholls, A.; Regalbuto, J., Synthesis of ultrasmall, homogeneously alloyed, bimetallic nanoparticles on silica supports. *Science* **2017,** *358* (6369), 1427-1430.
(16) Wang, L.; Yang, R. T., New sorbents for hydrogen storage by hydrogen spillover–a review. *Energy & Environmental Science* **2008,** *1* (2), 268-279.
(17) Barrett, E. P.; Joyner, L. G.; Halenda, P. P., The determination of pore volume and area distributions in porous substances. I. Computations from nitrogen isotherms. *Journal of the American Chemical Society* **1951,** *73* (1), 373-380.
(18) Larson, D.; Prosa, T.; Ulfig, R.; Geiser, B.; Kelly, T., *Local Electrode Atom Probe Tomography: A User's Guide*. 1st ed.; Springer: New York: 2013.
(19) Reddy, G. K.; Ling, C.; Peck, T. C.; Jia, H., Understanding the chemical state of palladium during the direct NO decomposition–influence of pretreatment environment and reaction temperature. *RSC Advances* **2017,** *7* (32), 19645-19655.
(20) Verberne, R.; Saxey, D. W.; Reddy, S. M.; Rickard, W. D.; Fougerouse, D.; Clark, C., Analysis of natural rutile (TiO_2) by laser-assisted atom probe tomography. *Microscopy and Microanalysis* **2019,** *25* (2), 539-546.
(21) Hatzoglou, C.; Rouland, S.; Radiguet, B.; Etienne, A.; Costa, G. D.; Sauvage, X.; Pareige, P.; Vurpillot, F., Preferential evaporation in atom probe tomography: An analytical approach. *Microscopy and Microanalysis* **2020,** *26* (4), 689-698.
